# Expression of ganglioside GD2, reprogram the lipid metabolism and EMT phenotype in bladder cancer

**DOI:** 10.18632/oncotarget.21038

**Published:** 2017-09-16

**Authors:** Venkatrao Vantaku, Sri Ramya Donepudi, Chandrashekar R. Ambati, Feng Jin, Vasanta Putluri, Khoa Nguyen, Kimal Rajapakshe, Cristian Coarfa, Venkata Lokesh Battula, Yair Lotan, Nagireddy Putluri

**Affiliations:** ^1^ Department of Molecular and Cell Biology, Baylor College of Medicine, Houston, TX, USA; ^2^ Dan L. Duncan Cancer Center, Advanced Technology Core, Alkek Center for Molecular Discovery, Baylor College of Medicine, Houston, TX, USA; ^3^ Section of Molecular Hematology and Therapy, Department of Leukemia, and Department of Breast Medical Oncology, The University of Texas MD Anderson Cancer Center, Houston, TX, USA; ^4^ Department of Urology, University of Texas Southwestern, Dallas, TX, USA

**Keywords:** bladder cancer, ganglioside GD2, lipid metabolism, EMT

## Abstract

High-grade Bladder Cancer (BLCA) represents the most aggressive and treatment-resistant cancer that renders the patients with poor survival. However, only a few biomarkers have been identified for the detection and treatment of BLCA. Recent studies show that ganglioside GD2 can be used as cancer biomarker and/or therapeutic target for various cancers. Despite its potential relevance in cancer diagnosis and therapeutics, the role of GD2 is unknown in BLCA. Here, we report for the first time that high-grade BLCA tissues and cell lines have higher expression of GD2 compared to low-grade by high-resolution Mass Spectrometry. The muscle invasive UMUC3 cell line showed high GD2, mesenchymal phenotype, and cell proliferation. Besides, we have shown the cancer stem cells (CSC) property (CD44hiCD24lo) of GD2+ UMUC3 and J82 cells. Also, the evaluation of lipid metabolism in GD2+ BLCA cell lines revealed higher levels of Phosphatidylinositol (PI), Phosphatidic acid (PA), Cardiolipin (CL) and lower levels of Phosphatidylserine (PS), plasmenyl-phosphatidylethanolamines (pPE), plasmenyl-phosphocholines (pPC), sphingomyelins (SM), triglycerides (TGs) and N-Acetylneuraminic acid. These findings are significantly correlated with the tissues of BLCA patients. Based on this evidence, we propose that GD2 may be used as an effective diagnostic and therapeutic target for aggressive BLCA.

## INTRODUCTION

Bladder Cancer (BLCA) is one of the leading causes of cancer-related deaths in Western countries, and is three to four times more prevalent in males than females [1, 2]. The treatment and its efficacy in BLCA vary depending upon the clinical stage and associated risk factors.

Muscle invasive bladder cancer (MIBC), which is typically associated with relatively poor prognosis have a 5-year survival of ~50% but for those who have metastasized cancer the expected 5-year survival is only ~15% [3–7]. Cystectomy with neoadjuvant chemotherapy is the front line treatment for muscle invasive bladder cancer [6, 8–10]. Since it has very little chance of being curative, it is not a viable option for patients with highly metastatic BLCA. Standard care for metastatic BLCA is a multidrug chemotherapy regimen consisting of cisplatin, methotrexate, vinblastine and adriamycin. Although muscle-invasive tumors initially respond to cisplatin based combination chemotherapy, the development of drug resistance is a major problem, and disease progression in resistant tumors is rapid and uniformly fatal [11]. Thus, treatment options for muscle-invasive BLCA are limited and have not significantly improved in recent years. The identification of novel biomolecules and their associated mechanisms that mediate invasion and metastasis in muscle-invasive tumors is of prime importance for patients with aggressive forms of BLCA.

Glycosphingolipids (GSLs) are amphiphilic membrane lipids that are ubiquitously expressed on all animal cell membranes, where they are involved in cell adhesion and signal transduction [12–14]. Gangliosides are sialic acid bearing glycosphingolipids expressed on all vertebrate cell membranes [15] and are anchored to the plasma membrane through ceramide lipids. Among gangliosides, GD2 is known to be highly expressed in ectoderm origin tumors such as neuroblastoma, melanoma, T-cell leukemia [16–18] and breast cancer stem cells [19] whereas they are weakly expressed in their normal tissues [20, 21]. Therefore, GD2 is considered to be a cancer-associated antigen. GD2 and certain other gangliosides are thought as promising targets for cancer immunotherapy because their expression is restricted to malignant cells and they are accessible on the cell surface. Moreover based on the therapeutic potential, immunogenicity, expression degree and antigen-specific cells percentage, the GD2 is ranked 12^th^ in the list of 75 potential targets for anti-cancer therapy by National Cancer Institute (NCI) [22].

The knowledge about GD2 in BLCA is yet not known. Here we studied GD2 through mass spectrometry-based approach and identified it as a biomarker for high-grade aggressive BLCA. This article will provide an overview of (a) expression of GD2 in bladder cancer tissues and cell lines, (b) classes of lipids or lipid metabolism associated with GD2 which lead to aggressive tumor cells; (c) GD2-positive BLCA cells display molecular and functional properties of CSCs and EMT phenotype.

## RESULTS

### GD2 enriches in bladder cancer patients and cell lines

**Figure 1:**
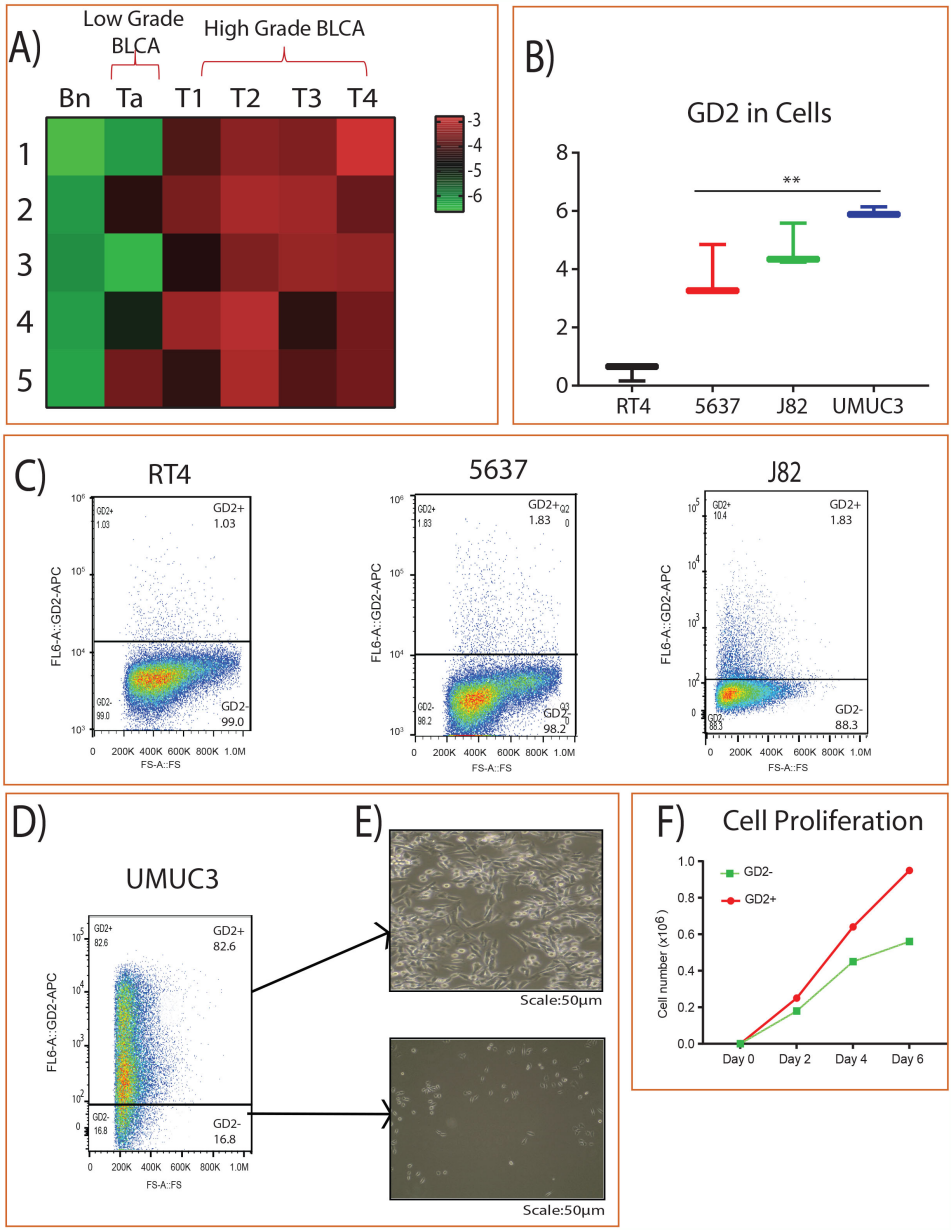
GD2 expression in BLCA **(A)** Heat map of GD2 expression across different stages of BLCA and the adjacent tissues. Columns represent individual tissue samples and rows represent GD2 expression. Shades of red and green represent higher and lower levels of GD2. **(B)** Box plot showing GD2 expression in BLCA cell lines by mass spectrometry. **(C)** BLCA (RT4, 5637, J82) cells were stained with anti-GD2 antibody and analyzed on FACS AriaII flow cytometer. GD2^+/–^ gates were drawn based on unstained and single stained controls FSC, forward scatter. **(D-E)** GD2^+/–^ UMUC3 cells were sorted and cultured in cell culture dishes for 4 days. (Scale bars: 50 μm). **(F)** 2 × 10^4^ GD2^+/–^ UMUC3 cells were grown in 6-well cell culture dishes in triplicate. Total cells were counted on days 2, 4, and 6 using a Vi-CELL (Beckman Coulter) cell counter (^*^ indicates p<0.05; ^**^ indicates p<0.001).

We analyzed the expression of GD2 in BLCA patients by using high-resolution mass spectrometry and identified its higher expression in high grade BLCA compared to adjacent benign and low grade BLCA (Figure 1A). Furthermore, muscle invasive /high-grade metastatic BLCA cell lines (UMUC3 and J82) also showed higher expression of GD2 compared to low grade/ non-muscle invasive aggressive BLCA cell lines (RT4 and 5637) (Figure 1B). In addition, we also used flow cytometry to check the expression of GD2 in BLCA cell lines and again found that the muscle invasive UMUC3 cell lines showed very high levels of GD2 [GD2+ (82.6%) and GD2– (16.8%)] than other BLCA cell lines (Figure 1C-1D). GD2+ UMUC3 cells were then isolated using FACS AriaII flow cytometer and displayed mesenchymal properties, whereas conversely, the GD2– cells displayed epithelial morphology (Figure 1E). Moreover, the GD2+ UMUC3 cells proliferated more rapidly than the GD2– UMUC3 cells (Figure 1F).

### GD2+ show CD44 high CD24 low population in BLCA cell lines

**Figure 2:**
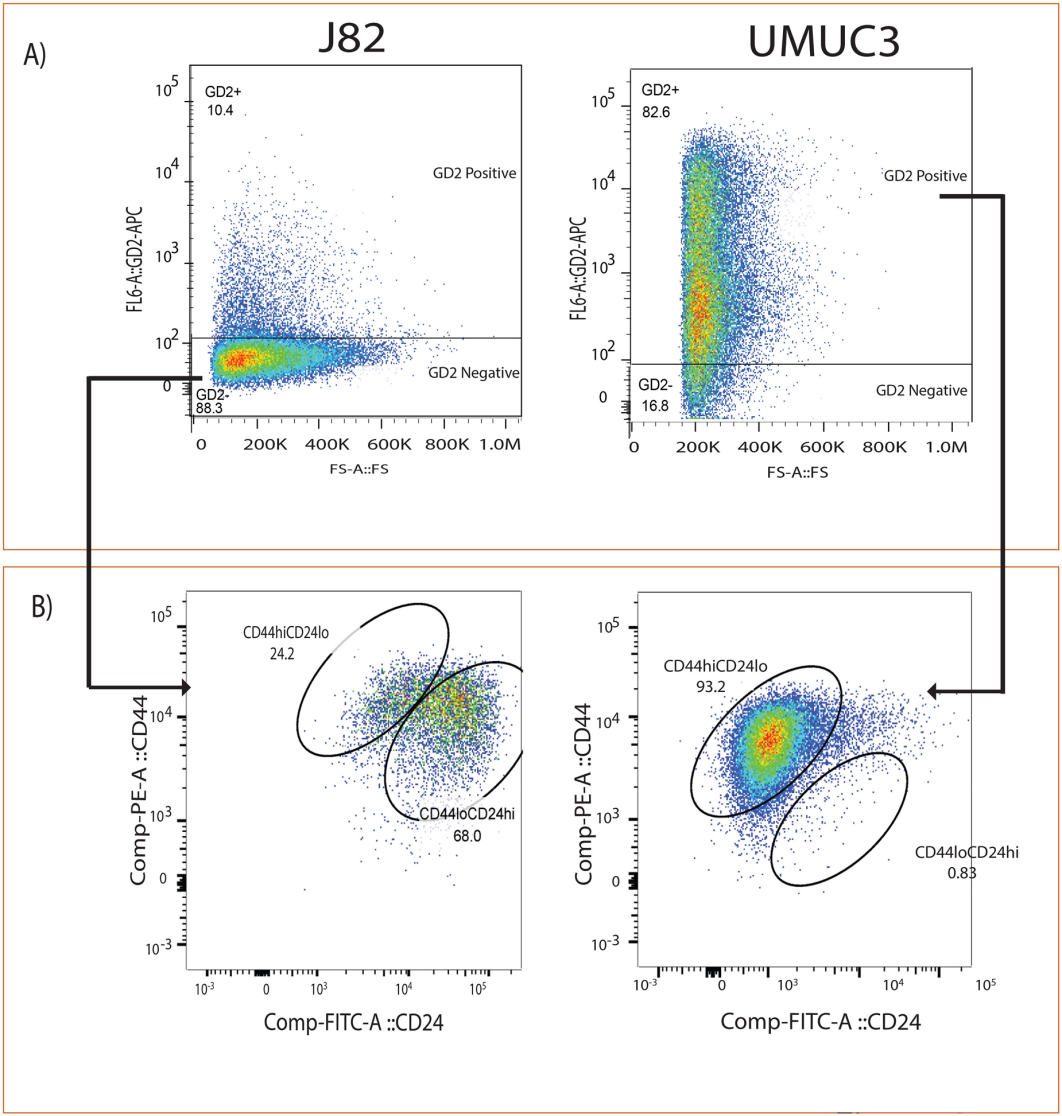
GD2 identifies CD44hiCD24lo in bladder cancer cells **(A)** Flow cytometry analysis of J82 and UMUC3 cells stained with anti-GD2-APC. **(B)** Anti-CD44-PE and anti-CD24-FITC antibodies cells were electrically gated on GD2+/– cells and displayed in a pseudo color dot plot with CD44 on the y-axis and CD24 on the x-axis using Flow Jo data analysis software.

Given that GD2, similar to previously reported CD44 and CD24 cell surface markers in other cancers [23], is able to separate cancer cells into two populations with differing tumor-initiating potential we hypothesize that GD2 would express CD44hiCD24lo cancer cell fraction. To examine this, we analyzed the expression of CD44hi CD24lo in GD2+/- UMUC3 and J82 cell lines (Figure 2A), and found that GD2+ UMUC3 cells show 93% of CD44hi CD24 lo, whereas GD2- J82 cells show less than 24% of CD44hi CD24lo (Figure 2B). Our results revealed that the UMUC3 cells demonstrate positive for GD2 and CD44 but negative for CD24, which suggested the existence of CSC phenotype.

### The altered lipid metabolism in GD2+ muscle invasive BLCA cell lines and tissues of high grade BLCA patients

**Figure 3:**
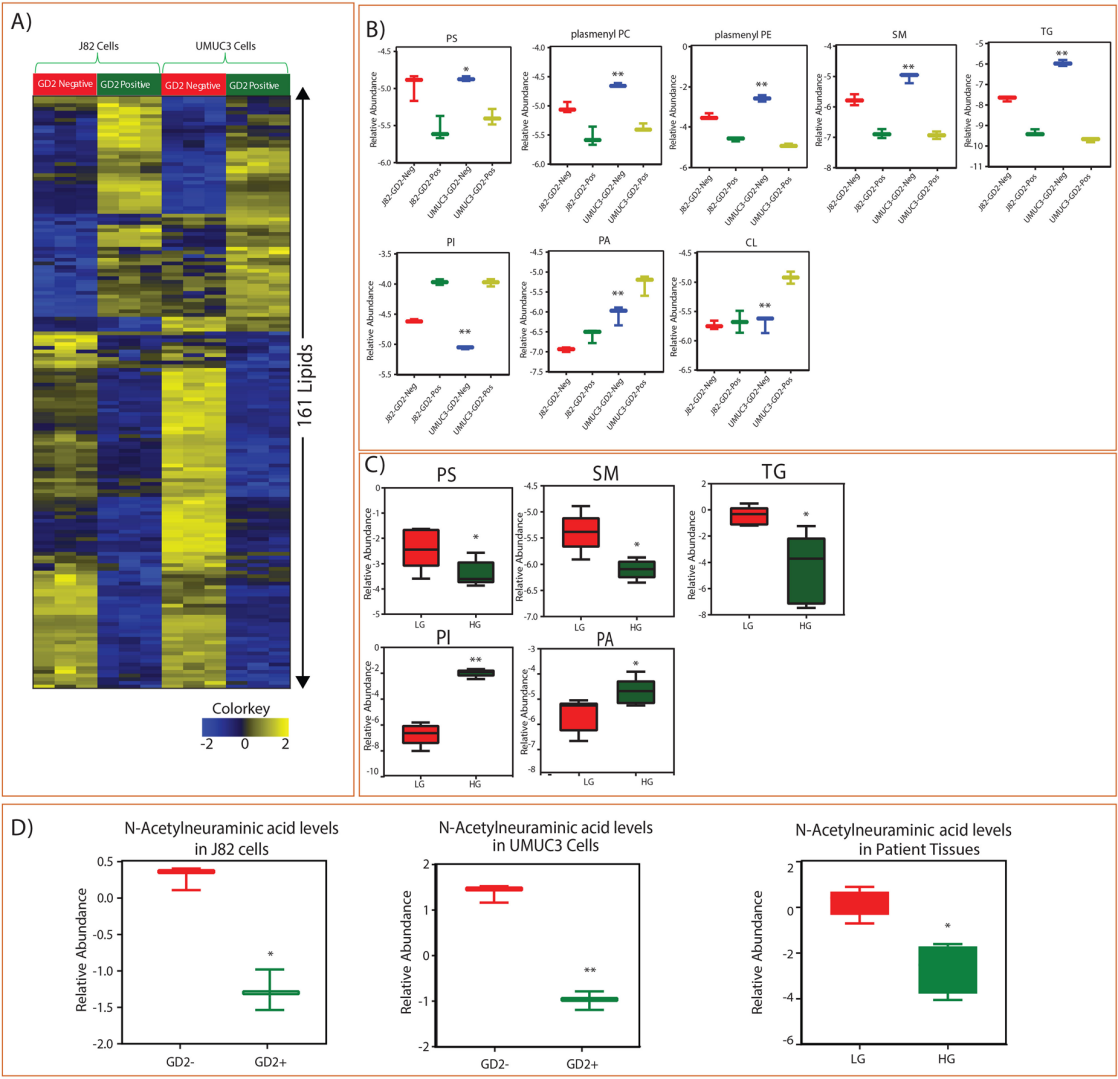
Identified lipid class alterations in GD2 +/- BLCA cell lines (J82 and UMUC3) and BLCA tissues **(A)** Heat map of GD2+/- sorted cells altered lipids from J82 and UMUC3 cell lines. Columns represent individual samples, and rows represent distinct lipids. Shades of yellow and blue represents higher and lower levels of lipids, relative to the median metabolite levels respectively (FDR<0.25). **(B)** Box plots are showing altered lipid classes in BLCA GD2+/- J82 and UMUC3 cell lines. **(C)** Box plots showing altered lipid classes in BLCA patients (LG= low grade, HG= high grade. **(D)** Box plots showing N-Acetylneumeric acid levels in cell lines and BLCA tissues (^*^ indicates p<0.05; ^**^ indicates p<0.001).

Since we observed differences in cell proliferation potential between GD2+ and GD2– populations, we investigated the lipid alterations in GD2+ and GD2– cells of muscle invasive J82 and UMUC3 cell lines. The analysis showed the alterations in the important lipid classes and identified 161 individual lipid species were altered between GD2+ and GD2– in both cell lines (Figure 3A). We have identified that GD2+ cells have lower levels of phosphatidylserine (PS), plasmenyl-phosphatidylethanolamines (pPE), plasmenyl-phosphocholines (pPC), sphingomyelins (SM) tricylglycerols (TGs) and higher levels of Phosphatidylinositol (PI), Phosphatidic acid (PA), and Cardiolipin (CL) (Figure 3B). These results correlate with tissues of BLCA patients, which also show low levels of PS, SM and TG and high levels of PI and PA class of lipids in high-grade BLCA patients compared to low-grade (Figure 3C). Further, a key metabolite N-Acetylneuraminic acid (NANA), which is a precursor for the biosynthesis of GD2, was low in high grade BLCA patients and GD2+ cells than low grade and GD2- suggesting its utilization in aggressive BLCA. Also we observed that NANA is low in cancer when compared to benign (Supplementary Figure 2).

### GD2+ cells EMT phenotype correlating with high grade BLCA patients

**Figure 4:**
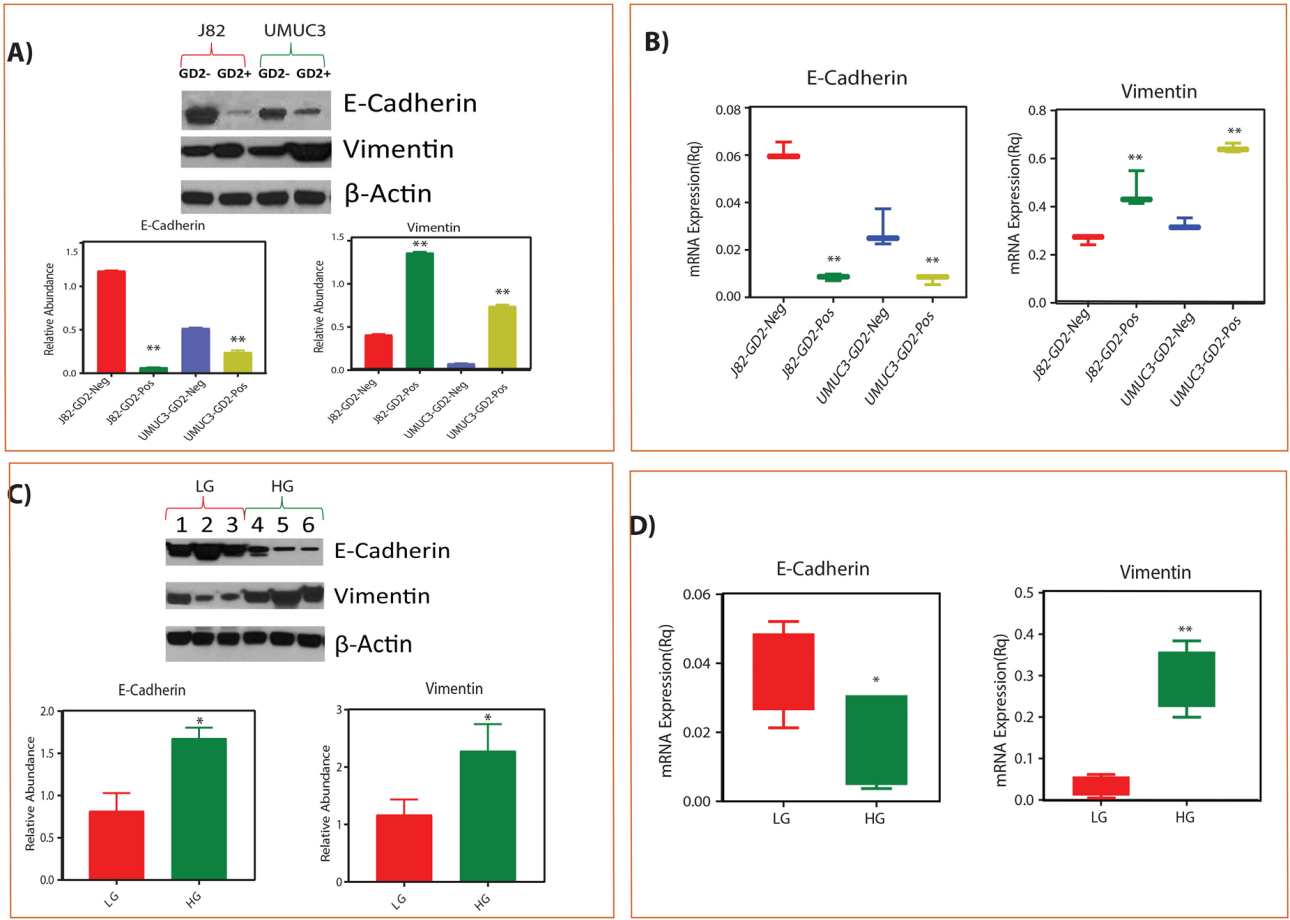
Correlation of GD2 +/- EMT phenotype with low grade and high grade tissues of BLCA patients **(A-B)** Protein and mRNA expression of EMT markers in GD2 +/- of J82 and UMUC3 cells. **(C-D)** Protein and mRNA expression of EMT markers in low grade and high-grade BLCA tissues respectively (^*^ indicates p<0.05; ^**^ indicates p<0.001).

To test the EMT phenotype of GD2+ cells, we performed Real Time-PCR and western blot with known EMT markers i.e E cadherin (epithelial) and vimentin (mesenchymal) and correlated with low and high grade BLCA tissues. We observed that the mesenchymal marker vimentin expression was high whereas epithelial marker E-cadherin expression was low in high-grade compared to low-grade BLCA tissues (Figure 4A-4B). We also observed high vimentin and low E cadherin expression in GD2+ cells and low vimentin and high E cadherin expression in GD2- cells supporting that GD2+ cells behave like high grade and GD2^-^ cells like low grade BLCA (Figure 4C-4D).

### Inhibition of GD2 switches the lipid metabolism and EMT

**Figure 5:**
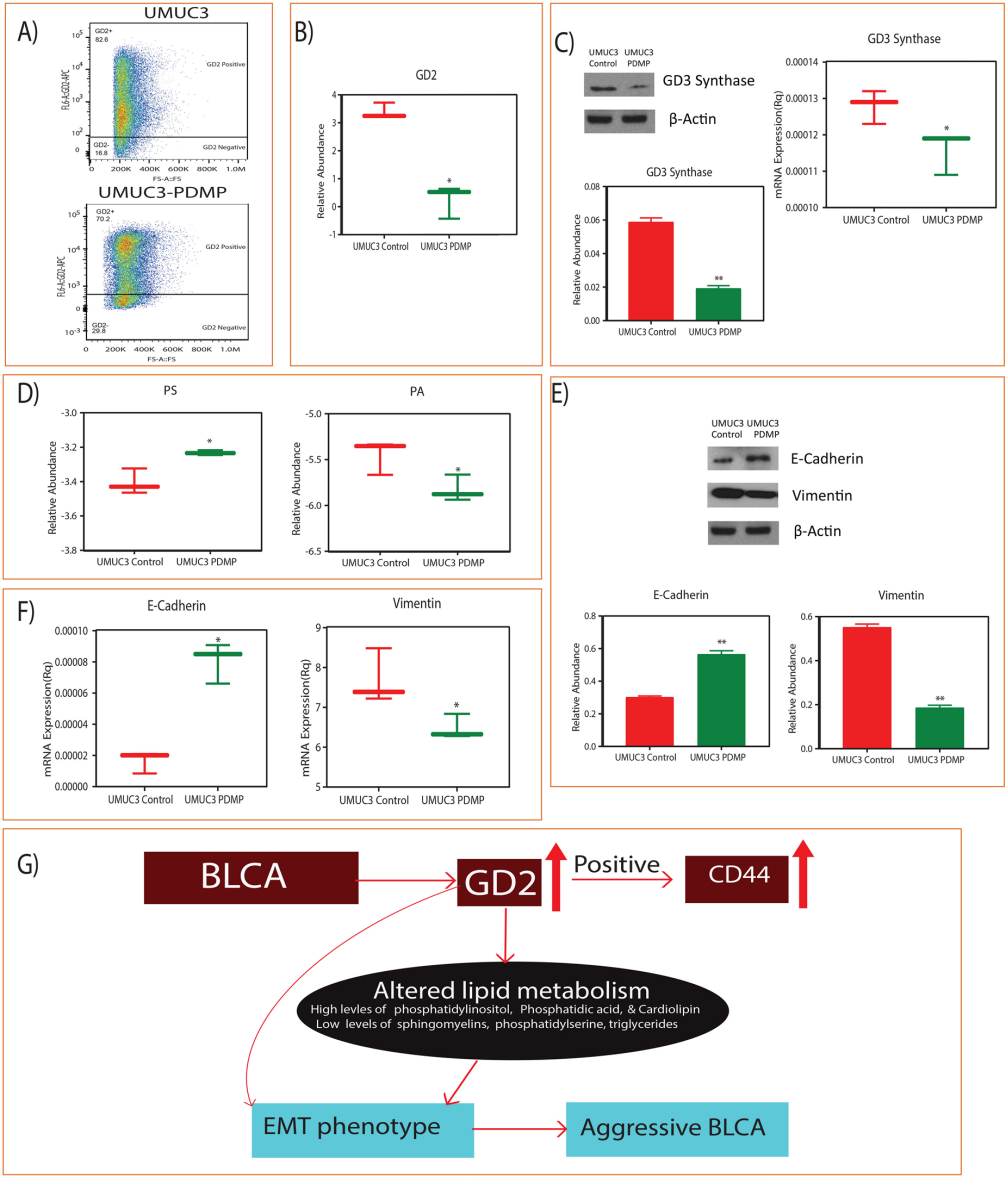
Inhibition of GD2 synthesis and switching of lipid classes and EMT phenotype **(A)** Flow cytometry analysis shows the reduction of GD2 upon 5 μM D-PDMP for 72hours. **(B)** Reduced GD2 levels measured by LC-MS. **(C)** Immunoblot and qPCR shows the reduced levels of GD3 synthase and their quantification. **(D)** Switching of PS and PA lipid classes. (**E** and **F**) Immunoblot and qPCR analysis of E cadherin and vimentin levels upon GD2 inhibition for 72 hours. **(G)** Working model of GD2 in high grade BLCA development (^*^ indicates p<0.05; ^**^ indicates p<0.001).

We further evaluated the GD2 role on lipid metabolism and EMT phenotype by inhibiting the GD2 synthesis by PDMP and found significant reduction in the synthesis of GD2 by flow cytometry and mass-spectrometry respectively (Figure 5A and 5B). We further checked the GD3 synthase, a major regulatory enzyme for the synthesis of GD2 in UMUC3 BLCA cells and found it to be inhibited by PDMP (Figure 5C). Interestingly, these low GD2 synthesized UMUC3 BLCA cells shows the switching of the PS and PA lipid class (Figure 5D) and EMT (Figure 5E and 5F) phenotype i.e, upregulation of E-cadherin and down regulation of vimentin.

## DISCUSSION

The Disialoganglioside GD2 is synthesized in the endoplasmic reticulum and Golgi's apparatus by an enzyme GD3 synthase, which is then transferred to the outer layer of the plasma membrane, where it is involves in cell-to-cell adhesion and signal transduction. It also plays a crucial role in physiological as well as pathological processes, such as cancer by driving proliferation, neoangiogenesis, immune-escape and invasion [24–26]. In this study, we identified the ganglioside GD2 expression in low-grade and high-grade BLCA patients and cell lines, and global lipidomic profile in GD2+ muscle invasive BLCA cell lines by using the high-resolution mass spectrometry. The BLCA patient tissues showed higher expression of GD2 compared to adjacent benign tissues. Of note, high-grade BLCA cell lines displayed higher expression of GD2 than low-grade BLCA cell lines. Specifically, GD2+ cells displayed stem cell properties similar to those of the CD44hiCD24lo population.

The high grade BLCA cell lines (J82, and UMUC3) also displayed a higher percentage of GD2+ cells than the low-grade cell lines (RT4) which is consistent with the clinical observation of the patients with more aggressive high grade BLCA. The mRNA and protein analysis of GD2+ cells showed high expression of the mesenchymal marker vimentin and downregulation of the epithelial marker E-cadherin, further supporting the mesenchymal nature of the GD2+ cells compared to GD2- cells. This is substantiated by the observation that GD2 expression is correlated with CD44 expression. The CD44 and Hyaluronan Mediated Motility Receptor (RHAMM) regulate the growth of bladder cancer cells [27]. Several studies have evidenced that GD2-specific antibodies inhibit tumor growth without the involvement of the immune system. The GD2+ muscle invasive BLCA cells mesenchymal nature indicates that the differentiated cancer cells acquire stem cell capacity through dedifferentiation, probably via EMT. In breast cancer, it has been reported that transformed HMLER cells could generate CD44hiCD24lo cells from CD44loCD24hi cells and vice versa, suggesting a spontaneous generation of stem-like cells from more differentiated cells [28].

Metabolic reprogramming is now a firmly established hallmark of cancer [29]. An implications of carbohydrates [30, 31] and amino acids [32, 33] metabolism in EMT phenotype acquisition has been well reported. The alterations in the lipid associated metabolic pathways encountered in tumors are now well recognized in recent years [34, 35]. Despite their role in a variety of processes of cancer development very little is known about the impact of lipid metabolism during EMT phenotype. In this study we identified that GD2+ BLCA cells reprogram the metabolism of the lipids that are correlated with high-grade BLCA and EMT. The lipid signatures of UMUC3 and J82 GD2+ cells unravels the presence of high levels of PI, PA and CL and low levels of phosphatidylserine (PS), plasmenyl-phosphatidylethanolamines (pPE), plasmenyl-phosphocholines (pPC), Sphingomyelins (SM) and triylglycerols (TGs) indicating the high dependence of invasive stage tumors on the phospholipids, especially PI, PA and CL. Earlier studies show that these phospholipid classes have prominent roles in tumor development. PIs are known for their involvement in intracellular signal transduction [36, 37]. In particular, the PI3-kinase pathway, which regulates many cellular functions, including lipid metabolism, is frequently repressed or activated in breast cancer [38, 39]. Accumulation of PI could affect not only cellular membrane fluidity but also the activity of PI3K signaling pathway [40–43]. Unlike our results, the lipid metabolism of Myc-induced lymphoma is characterized by reduced PI [44]. phosphatidic acid (PA) is synthesized from phosphotidylcholine (PC), a major component of cell membrane, by an enzyme Phospholipase D (PLD). Many evidences show that PLD has a role in cell migration which is key to cell invasion and metastasis [45, 46] and active PLD enhances lymphoma cell metastasis [47], where as the inactivation of PLD inhibited the metastasis, MMP-2 expression in glioma cell invasion [48]. The PLD promote invasion and metastasis possibly by producing more phosphatidic acid. Another phospholipid cardiolipin, found exclusively in mitochondrial membranes is intimately involved in mitochondrial functionality and membrane integrity [49]. An abnormal increase of cardiolipin in GD2+ needs to be further investigated for its role in cancer progression. Interestingly, N-Acetylneuraminic acid, a major sialic acid of gangliosides was found to be low in high grade BLCA patients and also in GD2+ cells, unlike other cancers such as head and neck cancer and non-small cell lung cancer where they are found to be very high in advanced cancer stages [50, 51]

The functional role of GD2 in BLCA is still not clear. New research suggests that gangliosides are widely expressed on tumor cells, and may help them escape from immune cells within the tumor microenvironment [21, 52, 53]. This is supported by several recent reports that gangliosides including GD1a, GD1b, GD3, and GM3 help tumors evade the immune attack by inducing apoptosis in immune cells, including T and NK cells [54, 55]. This is because the metastatic cancer cells have to travel a long distance to find a suitable microenvironment to initiate secondary tumors and that these cells have to overcome a complex immune system to reach their destination. Hence, the immunosuppressive function of GD2 may be critical for their metastatic ability. High GD2 expression has already been reported in breast cancer, melanomas, gliomas, and neuroblastomas [19, 56]. Anti-GD2 monoclonal antibodies therapeutic effects have been reported in neuroblastomas [57, 58]. Therefore, GD2 could be used as a potential marker for high grade BLCA and may be further developed as a therapeutic target.

In conclusion, to best of our knowledge, we have shown for the first time the GD2 expression in BLCA and also observed that its expression is higher in high-grade BLCA tissues and cell lines compared to low-grade. In addition, we have ascertained that GD2+ cells promote rapid BLCA growth and display cancer stem cell properties. Also, GD2+ cells, as well as high-grade BLCA patients, show altered lipid metabolism and EMT-positive phenotype. Thus, inhibiting the metabolism of these particular lipids can be beneficial for the treatment of high grade BLCA patients.

## MATERIALS AND METHODS

### Patient samples

A total of 25 pathologically-verified BLCA tissues and 5 adjacent benign tissues were obtained from the tumor banks of the University of Texas Southwestern Medical Center, TX, USA. We procured the sample cohort of low grade and high-grade BLCA tissues, which include early to late stages (Ta, T1, T2, T3 and T4), distant lymph nodes (N0, N1, N2, N3 and Nx), smoking status and survival status of BLCA (Supplementary Table 1).

### Cell culture

Bladder Cancer cell lines (RT4, 5637, J82, UMCU3) were obtained from American Type Culture Collection (ATCC, Manassas, VA, USA) and grown as per the vendor's instructions. All experiments were carried out within 6 months of procuring of these cells.

### Liquid chromatography- high-resolution mass spectrometry (LC-MS) for measurements of lipids

We used Shimadzu CTO-20A Nexera X2 UHPLC systems equipped with a degasser, binary pump, thermostatted auto sampler and a column oven for chromatographic separation. For lipid separation, 5 uL of lipid extract was injected to a 1.8 μm particle 50 × 2.1 mm Acquity HSS UPLC T3 column (Waters, Milford, MA) which was heated to 55°C. Acetonitrile/water (40:60, v/v) with 10 mM ammonium acetate was solvent A and acetonitrile/water/isopropanol (10:5:85 v/v/v) with 10 mM ammonium acetate was solvent B. For chromatographic elution we used a linear gradient over a 20 min total run time, with 60% Solvent A and 40% Solvent B gradient in the first 10 minutes. Then the gradient was ramped in a linear fashion to 100% Solvent B which was maintained for 7 minutes. After that the system was switched back to 60% Solvent B and 40% Solvent A for 3 minutes. The column was equilibrated for 3 min before the next injection and ran at a flow rate of 0.4 mL/min for a total run time of 20 min. The data acquisition of each sample was performed in positive ionization modes, using a Triple TOF 5600 equipped with a Turbo V™ ion source (AB Sciex, Concord, Canada). The column effluent was direct to the electrospray ionization source. The voltage of source was set to 5500 V for positive ionization and 4500 V for negative ionization mode, the declustering potential was set to 60 V, and the source temperature was set to 450°C for both modes. The curtain gas flow, nebulizer, and heater gas were set to 30, 40, and 45 units, respectively. The instrument performed one TOF MS survey scan (150 ms) and 15 MS/MS scans with a total duty cycle time of 2.4 s. The mass range in both modes was 50-1200 m/z. We controlled the acquisition of MS/MS spectra using the data-dependent acquisition (DDA) function of the Analyst TF software (AB Sciex, Concord, Canada) with the following parameters: dynamic background subtraction, charge monitoring to exclude multiply charged ions and isotopes, and dynamic exclusion of former target ions for 9s. The rolling collision energy spread was set whereby the software calculated the collision energy value to be applied as a function of m/z. Mass accuracy was maintained with an automated calibrant delivery system interfaced to the second inlet of the DuoSpray source.

### GD2 measurements

As described above, we used the TripleTOF 5600 equipped with a Turbo VTM ion source (AB Sciex, Concord, Canada) and double charged ion was used for the quantification (Supplementary Figure 1). Negative mode was used. A HPLC column, 3.5 μm particle 100 × 4.6 mm XBridge Amide column (Waters, Milford, MA) was used in this experiment. Mobile phase A is 20 mM ammonium phosphate in 95% water and 5% acetonitrile with pH 9.0 and MP B is 100% acetonitrile. The gradient was set at 85 % B at time 0, and then decreased to 30% at 16 min, then 2% at 24 min. After that, the gradient was kept at 2% B for two minutes, and then ramped to 85% until 36 min. One TOF-MS and fifteen product ion scans were performed during one cycle of 600 ms. TOF mass range was set from 50 to 900. The tissue and cell samples were

### Flow cytometry

Single color staining of all BLCA cell lines (RT4, 5637, J82, UMCU3) was performed to analyze the expression of GD2. Triple-color staining of J82 and UMUC3 cells was performed with conjugated antibodies GD2, CD44, and CD24 to test the cancer stem cell properties as follows. Cells were detached with trypsin, washed twice in PBS, then stained with anti GD2-APC, anti-CD24-FITC and anti-CD44-PE (Biolegend Antibodies) using 5 μl of antibody per 10^6^ cells, and incubated in ice for 15 min. Following incubation, cells were washed once with PBS. The 20 × 10^6^ cells were resuspended in 500ul of PBS and sorted using a FACS AriaII flow cytometer (BD) equipped with a 130μm nozzle running BD FACS Diva 8.0.1 software. Further analyses were performed with FlowJo software (Tree Star Inc). Live cells were gated on the basis of forward and side scatter area to reduce background noise, single cells were selected based on forward scatter (pulse area versus height) and side scatter (pulse width versus height) parameters, and dead cells were excluded based on DAPI uptake. For multicolor compensation and gating unstained, single-color and appropriate FMO controls were used.

### Inhibition of GD2 synthesis

To inhibit the GD2, cultured UMUC3 BLCA cells were treated with 5 μM D-PDMP (DL-threo-1-phenyl-2- decanolylamine-3-morpholino-1-propanol HCl) [59] (Millipore) for 72 hours. Expression of GD2 was measured by flow cytometry and mass spectrometry. DMSO was used as a control solvent.

## SUPPLEMENTARY MATERIALS FIGURES AND TABLES




