# Correction: Expression of ganglioside GD2, reprogram the lipid metabolism and EMT phenotype in bladder cancer

**DOI:** 10.18632/oncotarget.27311

**Published:** 2019-11-26

**Authors:** Venkatrao Vantaku, Sri Ramya Donepudi, Chandrashekar R. Ambati, Feng Jin, Vasanta Putluri, Khoa Nguyen, Kimal Rajapakshe, Cristian Coarfa, Venkata Lokesh Battula, Yair Lotan, Nagireddy Putluri

**Affiliations:** ^1^ Department of Molecular and Cellular Biology, Baylor College of Medicine, Houston, TX, USA; ^2^ Dan L. Duncan Cancer Center, Advanced Technology Core, Alkek Center for Molecular Discovery, Baylor College of Medicine, Houston, TX, USA; ^3^ Section of Molecular Hematology and Therapy, Department of Leukemia, and Department of Breast Medical Oncology, The University of Texas MD Anderson Cancer Center, Houston, TX, USA; ^4^ Department of Urology, University of Texas Southwestern, Dallas, TX, USA

**This article has been corrected:** During the assembly of Figure 4A, the protein expression for E-Cadherin, Vimentin and ß-actin were presented incorrectly. In addition, in Figure 5E, the protein expression for Vimentin was also presented incorrectly. Using source samples data, corrected Figures 4A and 5E were generated and are shown below. The following figure legend additions were also made:

Additions to Figure 2 legends: The flow cytometry data for J82, and UMUC3 in Panel A is derived from the same experiment described under Figure 1 (Refer to the Flow Cytometry Methods), and was GD2 gated to obtain panel B.

Additions to Figure 5 legends: The flow cytometry data for UMUC3 (WT) shown in top portion of Panel A is derived from Figure 1. Flow cytometry analysis shows the reduction of GD2 in WT cells (and DMSO controls, data not shown) upon 5 µM D-PDMP for 72 hours.

Corrections in the results sections:

**GD2+ show CD44 high CD24 low population in BLCA cell lines**

Given that GD2, similar to previously reported CD44 and CD24 cell surface markers in other cancers [23], is able to separate cancer cells into two populations with differing tumor-initiating potential we hypothesize that GD2 would express CD44hiCD24lo cancer cell fraction. To examine this, we analyzed the expression of CD44hi CD24lo in GD2+/- UMUC3 and J82 cell lines (representative data in Figure 2A) in two biological replicates, and found that GD2+ UMUC3 cells show approximately 93% of CD44hi CD24 lo, whereas GD2- J82 cells (representative data in Figure 2B) show less than 24% of CD44hi CD24lo. Our results revealed that the UMUC3 cells demonstrate positive for GD2 and CD44 but negative for CD24, which suggested the existence of CSC phenotype.

Original article: Oncotarget. 2017; 8:95620–95631. 95620-95631. https://doi.org/10.18632/oncotarget.21038

**Revised Figure 4A:**
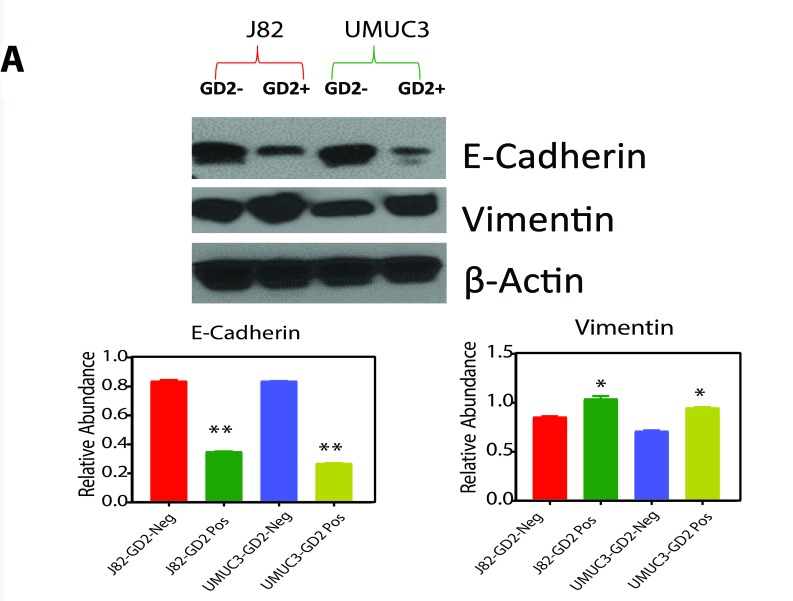
Protein expression of EMT markers in GD2 +/- of J82 and UMUC3 cells.

**Revised Figure 5E:**
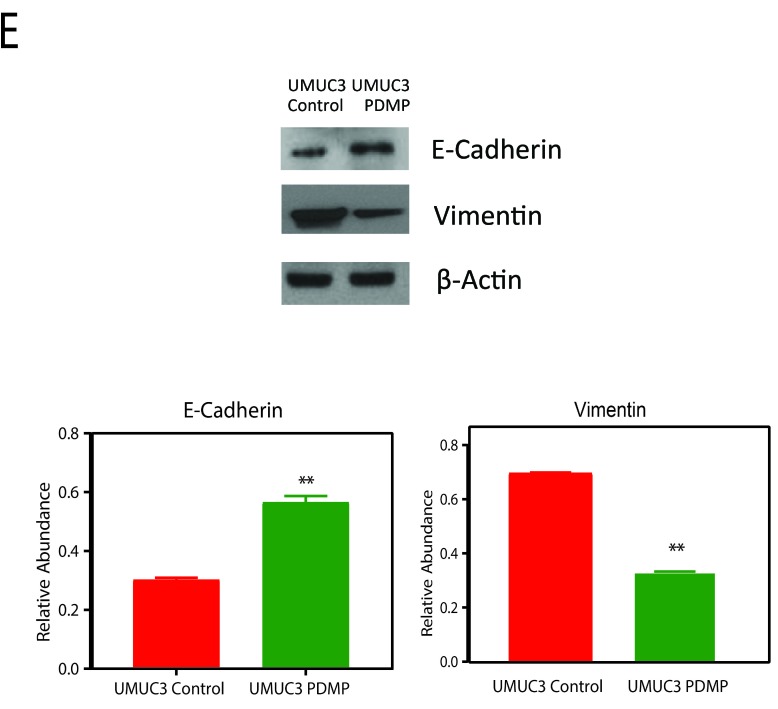
Immunoblot analysis of E cadherin and vimentin levels upon GD2 inhibition for 72 hours.

