# Supplementary material for: Expression of ganglioside GD2, reprogram the lipid metabolism and EMT phenotype in bladder cancer
Source: Oncotarget. 2017 Sep 16;8(56):95620–31. doi: 10.18632/oncotarget.21038 (PMC5707048; doi:10.18632/oncotarget.21038)
Supplement: Supplementary file 1 [file oncotarget-08-95620-s001.pdf]

Expression of ganglioside GD2, reprogram the lipid metabolism and EMT phenotype in bladder cancer

SUPPLEMENTARY MATERIALS

Sample preparation for mass spectrometry analysis

Samples were stored at -80°C until extraction. We used 50 mg of tissues for sample preparation. Lipids were extracted using a modified Bligh-Dyer method [1]. The extraction was carried out using 2:2:2 volume ratio of water/methanol/dichloromethane at room temperature after spiking internal standards (17:0 LPC, 17:0 PC, 17:0 PE, 17:0 PG, 17:0 ceramide, 17:0 SM, 17:0 PS, 17:0 PA, 17:0 TAG, 17:0 MAG, DAG 16:0/18:1, CE 17:0, and 17.0-20.4 PI) (Supplementary Table 2). The organic layer was collected and completely dried under nitrogen. Before MS analysis, the dried extract was resuspended in 100 µL of Buffer B (10:5:85 Acetonitrile/water/Isopropyl alcohol) containing 10mM NH_4OAc and subjected to LC/MS. The lipidome was separated using reverse-phase chromatography.

Reagents and Internal Standards

We used a high-performance Liquid Chromatography (LC) grade acetonitrile, and dichloromethane from Sigma (St. Louis, MO), isopropanol from Optima- Liquid Chromatography/Mass Spectrometry (LC/MS) Fisher (New Jersey, NJ), and methanol from J.T. Baker (Radnor, PA). We obtained water from a Millipore high purity water dispenser (Billerica, MA). We purchased the MS grade lipid standards from Avanti Polar Lipids (Alabaster, AL) (Supplementary Table 2).

Internal standards and quality controls

We prepared the lipid stock solutions by weighing an exact amount of the lipid internal standards in Chloroform/Methanol/H_2O resulting in a concentration of 1mg/mL and stored at -20 °C. We further diluted the stock solutions to 100pmol/uL by mixing appropriate volume of the internal standards LPC 17:0/0:0, PG 17:0/17:0, PE 17:0/17:0, PC 17:0/17:0, TAG 17:0/17:0/17:0, SM 18:1/17:0, MAG 17:0, DAG 16:0/18:1, CE 17:0, ceramide d 18:1/17:0, PA 17:0, PI 17:0/20:4, and PS 17:0/17:0. We used two kinds of controls to monitor the sample preparation and MS. To monitor instrument performance, we used 10 µL of a dried matrix-free mixture of the internal standards, reconstituted in 100 µL of buffer B (5% water, 85%Isopropanolol:10%Acetonitrile in 10mM NH_4OAc). To monitor the lipid extraction process, we used a standard pool of tissue samples from aliquots from the 165 samples. For quality control, we ran the pooled samples every day in the beginning, and at the end day. We also spiked the internal standards into test samples during the extraction process.

Data processing

We converted the raw data to mgf data format using proteo wizard software [2]. We used the NIST MS PepSearch Program to search the converted files against LipidBlast libraries [3, 4]. The *m/z* width was determined by the mass accuracy of internal standards and was set 0.001 for positive mode and 0.005 for a negative mode with an overall mass error of less than 2 parts per million. The minimum match factor used was set to 400. The MS/MS identification results from all the files were combined using an in-house software tool to create a library for quantification. All raw data files were searched against this library of identified lipids with mass and retention time using Multiquant 1.1.0.26 (ABsciex, Concord, Canada) [5]. We used MS/MS data as an intermediary step to help with identification, but quantification was done using MS1 data only. Relative abundance of peak spectra was used for the analyses. The lipids which were identified in both positive and negative ion modes were initially analyzed separately for their relationship with outcome to ensure persistent results. Identified lipids by type of adduct and retention times in positive and negative modes are show in Supplementary Table 3. As the relationship with outcome was not different in such lipids by ion modes, the values from positive mode were used in the final analysis. For lipid features with multiple adducts, the sum of spectral peaks from different adducts was used for the corresponding lipid. Identified lipids were quantify by normalizing against their respective internal standard. Quality Control samples were used to monitor the overall quality of the lipid extraction and mass spectrometry analyses [6-12].

QPCR and western blot analysis

Muscle invasive (J82 and UMUC3) bladder cancer cell line were procured from American Type Culture Collection (ATCC, Manassas, VA, USA) and maintained

as per ATCC recommendations. Short Tandem Repeat (STR) Profiling of these cell lines were reported earlier [13, 14]. mRNA expression was measured by QPCR by the following steps. RNA was extracted by RNeasy Mini Kit (Qiagen) from washed cell pellets and clinically confirmed bladder cancer low grade Ta and high grade T4 tissues. The quality and quantity were verified by measuring the absorbance at 260 and 280 nm. 1st strand cDNA was synthesized by using qScript™ cDNA SuperMix, (Quanta Biosciences), and qPCR was performed on StepOnePlus, (Applied Biosystems) using the SYBR green Master Mix and gene specific primers (Supplementary Table 4). The 2-ΔΔCt method was used to calculate relative changes in mRNA levels, GAPDH was used as an internal control. For protein expression analysis, western blot was performed. Protein was extracted from both cell lines and tissues using RIPA buffer (Sigma-Aldrich) with added protease and phosphatase inhibitor cocktail (Thermo scientific), and run on 4–20% Mini-PROTEAN® TGX™ gels (BIORAD). Gels were blotted on PVDF membranes. Membrane was blocked with 5% skim milk in TBST, Incubation with primary antibodies was performed overnight in 1% milk in TBST (Tris-buffered saline and Tween 20). HRP-conjugated secondary antibodies were detected using the SuperSignal West Pico Chemiluminescent Substrate (Pierce Rockford, IL). All kits were used according to the manufacturer's instructions

REFERENCES

1. Bligh EG, Dyer WJ. A rapid method of total lipid extraction and purification. Can J Biochem Physiol. 1959; 37: 911-7. https://doi.org/10.1139/o59- 099.
2. Chambers MC, Maclean B, Burke R, Amodei D, Ruderman DL, Neumann S, Gatto L, Fischer B, Pratt B, Egertson J, Hoff K, Kessner D, Tasman N, et al. A cross-platform toolkit for mass spectrometry and proteomics. Nat Biotechnol. 2012; 30: 918-20. https://doi.org/10.1038/ nbt.2377.
3. Kind T, Meissen JK, Yang D, Nocito F, Vaniya A, Cheng YS, Vandergheynst JS, Fiehn O. Qualitative analysis of algal secretions with multiple mass spectrometric platforms. J Chromatogr A. 2012; 1244: 139-47. https://doi.org/10.1016/j.chroma.2012.04.074.
4. Meissen JK, Yuen BT, Kind T, Riggs JW, Barupal DK, Knoepfler PS, Fiehn O. Induced pluripotent stem cells show metabolomic differences to embryonic stem cells in polyunsaturated phosphatidylcholines and primary metabolism. PLoS One. 2012; 7: e46770. https://doi.org/10.1371/journal.pone.0046770.
5. Ejsing CS, Duchoslav E, Sampaio J, Simons K, Bonner R, Thiele C, Ekroos K, Shevchenko A. Automated identification and quantification of glycerophospholipid molecular species by multiple precursor ion scanning. Anal Chem. 2006; 78: 6202-14. https://doi.org/10.1021/ac060545x.
6. Jung HR, Sylvanne T, Koistinen KM, Tarasov K, Kauhanen D, Ekroos K. High throughput quantitative molecular lipidomics. Biochim Biophys Acta. 2011; 1811: 925-34. https://doi.org/10.1016/j.bbalip.2011.06.025.
7. Putluri N, Shojaie A, Vasu VT, Nalluri S, Vareed SK, Putluri V, Vivekanandan-Giri A, Byun J, Pennathur S, Sana TR, Fischer SM, Palapattu GS, Creighton CJ, et al. Metabolomic profiling reveals a role for androgen in activating amino acid metabolism and methylation in prostate cancer cells. PLoS One. 2011; 6: e21417. https://doi.org/10.1371/journal.pone.0021417.
8. Putluri N, Shojaie A, Vasu VT, Vareed SK, Nalluri S, Putluri V, Thangjam GS, Panzitt K, Tallman CT, Butler C, Sana TR, Fischer SM, Sica G, et al. Metabolomic profiling reveals potential markers and bioprocesses altered in bladder cancer progression. Cancer Res. 2011; 71: 7376-86. https://doi.org/10.1158/0008-5472.CAN-11-1154.
9. Kaushik AK, Shojaie A, Panzitt K, Sonavane R, Venghatakrishnan H, Manikkam M, Zaslavsky A, Putluri V, Vasu VT, Zhang Y, Khan AS, Lloyd S, Szafran AT, et al. Inhibition of the hexosamine biosynthetic pathway promotes castration-resistant prostate cancer. Nat Commun. 2016; 7: 11612. https://doi. org/10.1038/ncomms11612.
10. Park JH, Vithayathil S, Kumar S, Sung PL, Dobrolecki LE, Putluri V, Bhat VB, Bhowmik SK, Gupta V, Arora K, Wu D, Tsouko E, Zhang Y, et al. Fatty acid oxidation-driven src links mitochondrial energy reprogramming and oncogenic properties in triple- negative breast cancer. Cell Rep. 2016; 14: 2154-65. https://doi.org/10.1016/j.celrep.2016.02.004.
11. von Rundstedt FC, Rajapakshe K, Ma J, Arnold JM, Gohlke J, Putluri V, Krishnapuram R, Piyarathna DB, Lotan Y, Godde D, Roth S, Storkel S, Levitt JM, et al. Integrative pathway analysis of metabolic signature in bladder cancer: a linkage to the Cancer Genome Atlas project and prediction of survival. J Urol. 2016; 195: 1911-9. https://doi. org/10.1016/j.juro.2016.01.039.
12. Yoon WH, Sandoval H, Nagarkar-Jaiswal S, Jaiswal M, Yamamoto S, Haelterman NA, Putluri N, Putluri V, Sreekumar A, Tos T, Aksoy A, Donti T, Graham BH, et al. Loss of nardilysin, a mitochondrial co-chaperone for alpha-ketoglutarate dehydrogenase, promotes mTORC1 activation and neurodegeneration. Neuron. 2017; 93: 115-31. https://doi.org/10.1016/j.neuron.2016.11.038.
13. Chiong E, Dadbin A, Harris LD, Sabichi AL, Grossman HB. The use of short tandem repeat profiling to characterize human bladder cancer cell lines. J Urol. 2009; 181: 2737-48. https://doi.org/10.1016/j.juro.2009.01.108.
14. Masters JR, Thomson JA, Daly-Burns B, Reid YA, Dirks WG, Packer P, Toji LH, Ohno T, Tanabe H, Arlett CF,

Kelland LR, Harrison M, Virmani A, et al. Short tandem repeat profiling provides an international reference standard for human cell lines. Proc Natl Acad Sci U S A. 2001; 98: 8012-7. https://doi. org/10.1073/pnas.121616198.

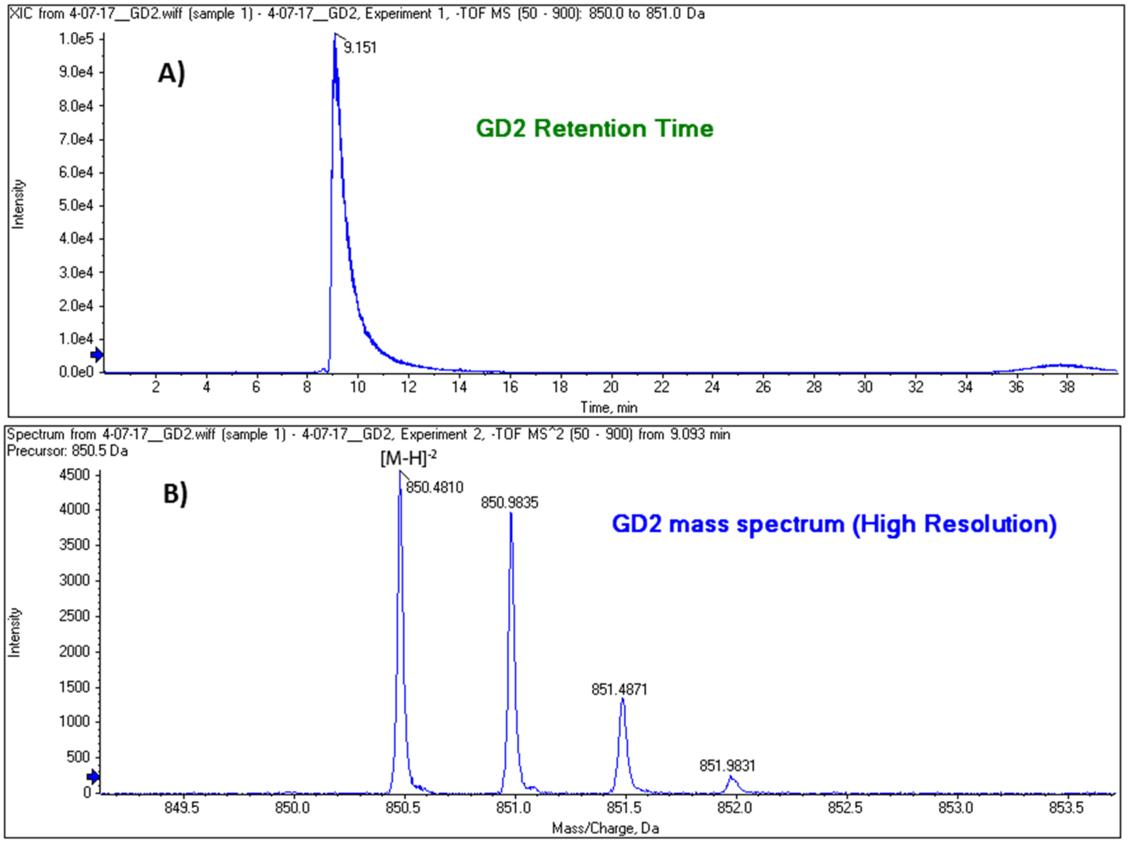

Supplementary Figure 1: GD2 identified on the Q-TOF negative electrospray ionization **(A)** extracted ion chromatography (EIC), **(B)** mass spectrum.

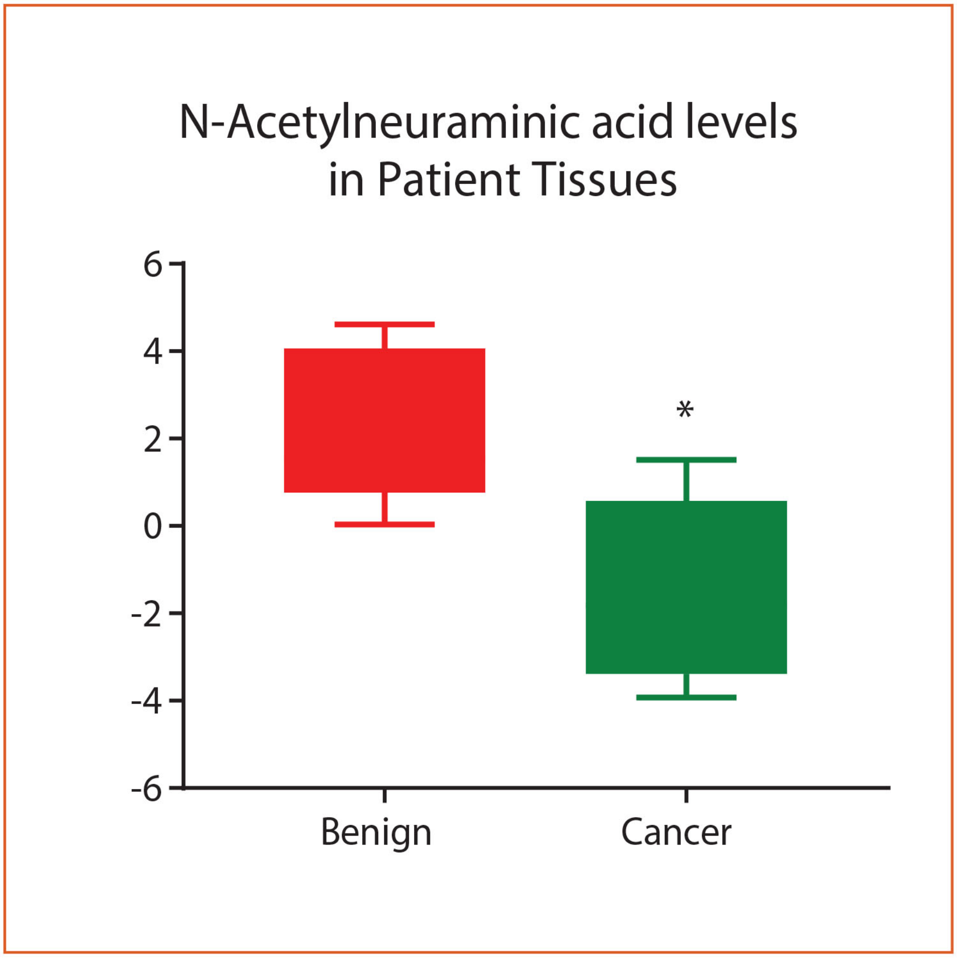

Supplementary Figure 2: N-Acetylneuraminic acid (NANA) levels in benign Vs cancer (* indicates p<0.05; ** indicates p<0.001).

Supplementary Table 1: Bladder cancer specimens used for this study [from UT Southwestern (UTSW)]

BLCA tissue samples	
	**BLCA patients characteristics**
**Stage**	Ta (n= 5; 20%)
	T1 (n=5; 20%)
	T2 (n=5; 20 %)
	T3 (n=5; 20 %)
	T4 (n=5; 20 %)
**Gender**	n= 23(92%; Male)
	n=02(8%; Female)
**Vital status**	n=18 (72%; Live)
	n=06(24%; Dead)
	n=01 (4%; Missing)
**Lymph node metastasis**	N0 (n=19; 76%)
	N1 (n=3; 12%)
	NX (n=1; 4%)
	N3 (n=1; 4%)
	Missing (n=1; 4%)
**Smoking status**	n=5 (20%; Never smoker)
	n=17 (68%; Ex-smoker)
	n=2 (8%; Current smoker)
	n=1 (4%; Missing)
**Cohorts**	UTSW (n= 25; 100%)
**Benign Tissue samples**	
**Benign Tissues**	n=5

Supplementary Table 2: Lipid internal Standards used for this study

1-heptadecanoyl-2-hydroxy-*sn*-glycero-3-phosphocholine (LPC) (17:0/0:0)
1,2-diheptadecanoyl-sn-glycero-3- Phosphatidylcholine (PC) (17:0/17:0)
1,2-diheptadecanoyl-sn-glycero-3- Phosphatidylethanolamine (PE) (17:0/17:0),
1,2-diheptadecanoyl-*sn*-glycero-3- Phosphatidylserine (PS) (17:0/17:0)
N-heptadecanoyl-D-*erythro*- sphingosylphosphorylcholine 17:0 (SM) (d18:1/17:0)
cholest-5-en-3ß-yl heptadecanoate 17:0 cholesteryl ester (CE)
1-palmitoyl-2-oleoyl-*sn*-glycerol 16:0-18:1 (DAG)
1-heptadecanoyl-rac-glycerol 17:0 (MAG)
1,2,3-triheptadecanoyl-glycerol Tri-heptadecanoate 17:0 (TAG)
N-heptadecanoyl-D-*erythro*-sphingosine C17 Ceramide (d18:1/17:0) (Cer-P)
1,2-diheptadecanoyl-*sn*-glycero-3-phosphate 17:0 (PA)
1,2-diheptadecanoyl-*sn*-glycero-3-phospho-(1'-*rac*-glycerol) 17:0 Phosphoglycerol (PG)
1-heptadecanoyl-2-(5Z,8Z,11Z,14Z-eicosatetraenoyl)-sn-glycero-3-phospho-(1'-myo-inositol) 17:0-20:4 Phosphatidylinositol (PI)

Supplementary Table 3: Identified lipids by type of adduct and retention time in positive and negative modes (Internal standards were indicated in green)

See Supplementary File 1

Supplementary Table 4: Gene-specific primers used for qPCR

	Primer sequence (5'-3')	
**Gene**	**Sense**	**Anti-sense**
GAPDH	GAGTCAACGGATTTGGTCGT	TTGATTTTGGAGGGATCTCG
E-CADHERIN	CGAGAGCTACACGTTCACGG	GGGTGTCGAGGGAAAAATAGG
VIMENTIN	AGCTAACCAACGACAAGCC	TCCACTTTGCGTTCAAGGTC
