# Supplementary material for: Expression of ganglioside GD2, reprogram the lipid metabolism and EMT phenotype in bladder cancer
Source: Oncotarget. 2017 Sep 16;8(56):95620–31. doi: 10.18632/oncotarget.21038 (PMC5707048; doi:10.18632/oncotarget.21038)
Supplement: Supplementary file 2 [file oncotarget-08-95620-s002.docx]

| **Supplementary Table 3:** Identified lipids by type of adduct and retention time in positive and negative modes (Internal standards were indicated in green). | | | | | | | | |
| --- | --- | --- | --- | --- | --- | --- | --- | --- |
| **Lipid Class** | **Carbon chain length** | **Double Bonds** | **Chain Category** | **Saturation of carbon** | **Lipid Name** | **Adducts** | **Retention Time** | **Method** |
| CE | 17 | 0 | <20 | Saturated | IS CE 17:0 | [M+NH4]+ | 6.81 | Positive |
| CE | 17 | 0 | <20 | Saturated | IS Ceramide | [M-H20]+ | 6.81 | Positive |
| TG | 15 | 0 | <20 | Saturated | IS D31-TAG | IS D31-TAG | 10.14 | Positive |
| DG | 17 | 0 | <20 | Saturated | IS d5-DAG | IS d5-DAG | 8.7 | Positive |
| DG | 34 | 1 | 20 to 40 | Monounsaturated | IS DG 34:1 | [M+NH4]+ | 9.7 | Positive |
| Lyso PC | 17 | 0 | <20 | Saturated | IS lysoPC 17:0 | [M+H]+ | 1.78 | Positive |
| MG | 34 | 0 | 20 to 40 | Saturated | IS MG 17:0 | [M+NH4]+ | 2.878 | Positive |
| PC | 34 | 0 | 20 to 40 | Saturated | IS PC 34:0 | [M+H]+ | 6.79 | Positive |
| PE | 34 | 0 | 20 to 40 | Saturated | IS PE 34:0 | [M+H]+ | 6.9 | Positive |
| PS | 34 | 0 | 20 to 40 | Saturated | IS PS 34:0 | [M+H]+ | 5.4 | Positive |
| SM | 35 | 1 | 20 to 40 | Monounsaturated | IS SM 35:1 | [M+H]+ | 5.7 | Positive |
| TG | 51 | 0 | >= 40 | Saturated | IS TG 51:0 | [M+NH4]+ | 10.74 | Positive |
| CE | 18 | 1 | < 20 | Monosaturated | CE(18:1) | [M+NH4]+ | 10.78 | Positive |
| CE | 18 | 2 | < 20 | Polysaturated | CE(18:2) | [M+NH4]+ | 10.56 | Positive |
| CE | 18 | 1 | < 20 | Monosaturated | N-(15Z-tetracosenoyl)-sphing-4-enine | [M+H]+ | 9.79 | Positive |
| CE | 18 | 0 | < 20 | Saturated | N-(hexadecanoyl)-sphinganine | [M+H]+ | 10.68 | Positive |
| DG | 24 | 0 | 20-40 | Saturated | DG 24:0 | [M+Li]+ | 10.05 | Positive |
| DG | 26 | 4 | 20-40 | Polysaturated | DG 26:4 | [M+Li]+ | 10.99 | Positive |
| DG | 30 | 0 | 20-40 | Saturated | DG 30:0 | [M+NH4]+ | 9.80 | Positive |
| DG | 32 | 1 | 20-40 | Monosaturated | DG 32:1 | [M+NH4]+ | 9.65 | Positive |
| DG | 32 | 1 | 20-40 | Monosaturated | DG 32:1 | [M+NH4]+ | 9.79 | Positive |
| DG | 32 | 2 | 20-40 | Polysaturated | DG 32:2 | [M+NH4]+ | 9.77 | Positive |
| DG | 33 | 1 | 20-40 | Monosaturated | DG 33:1 | [M+NH4]+ | 10.11 | Positive |
| DG | 34 | 0 | 20-40 | Saturated | DG 34:0 | [M+NH4]+ | 9.89 | Positive |
| DG | 34 | 1 | 20-40 | Monosaturated | DG 34:1 | [M+NH4]+ | 9.36 | Positive |
| DG | 34 | 3 | 20-40 | Polysaturated | DG 34:3 | [M+NH4]+ | 10.56 | Positive |
| DG | 35 | 1 | 20-40 | Monosaturated | DG 35:1 | [M+NH4]+ | 9.85 | Positive |
| DG | 35 | 2 | 20-40 | Polysaturated | DG 35:2 | [M+NH4]+ | 9.95 | Positive |
| DG | 36 | 1 | 20-40 | Monosaturated | DG 36:1 | [M+NH4]+ | 9.74 | Positive |
| DG | 36 | 1 | 20-40 | Monosaturated | DG 36:1 | [M+NH4]+ | 7.56 | Positive |
| DG | 36 | 2 | 20-40 | Polysaturated | DG 36:2 | [M+NH4]+ | 9.91 | Positive |
| DG | 36 | 3 | 20-40 | Polysaturated | DG 36:3 | [M+NH4]+ | 10.13 | Positive |
| DG | 38 | 1 | 20-40 | Monosaturated | DG 38:1 | [M+NH4]+ | 10.06 | Positive |
| DG | 38 | 2 | 20-40 | Polysaturated | DG 38:2 | [M+NH4]+ | 10.09 | Positive |
| DG | 38 | 3 | 20-40 | Polysaturated | DG 38:3 | [M+NH4]+ | 8.10 | Positive |
| DG | 38 | 3 | 20-40 | Polysaturated | DG 38:3 | [M+NH4]+ | 9.96 | Positive |
| DG | 38 | 4 | 20-40 | Polysaturated | DG 38:4 | [M+NH4]+ | 1.67 | Positive |
| DG | 40 | 1 | >= 40 | Monosaturated | DG 40:1 | [M+NH4]+ | 8.79 | Positive |
| DG | 40 | 1 | >= 40 | Monosaturated | DG 40:1 | [M+NH4]+ | 9.47 | Positive |
| DG | 40 | 2 | >= 40 | Polysaturated | DG 40:2 | [M+NH4]+ | 7.68 | Positive |
| DG | 40 | 2 | >= 40 | Polysaturated | DG 40:2 | [M+NH4]+ | 8.09 | Positive |
| DG | 40 | 3 | >= 40 | Polysaturated | DG 40:3 | [M+NH4]+ | 7.69 | Positive |
| DG | 40 | 4 | >= 40 | Polysaturated | DG 40:4 | [M+NH4]+ | 9.59 | Positive |
| DG | 42 | 0 | >= 40 | Saturated | DG 42:0 | [M+NH4]+ | 7.31 | Positive |
| DG | 42 | 1 | >= 40 | Monosaturated | DG 42:1 | [M+NH4]+ | 10.77 | Positive |
| DG | 42 | 2 | >= 40 | Polysaturated | DG 42:2 | [M+NH4]+ | 7.63 | Positive |
| DG | 44 | 1 | >= 40 | Monosaturated | DG 44:1 | [M+NH4]+ | 9.87 | Positive |
| DG | 44 | 2 | >= 40 | Polysaturated | DG 44:2 | [M+NH4]+ | 9.50 | Positive |
| lysoPC | 16 | 0 | < 20 | Saturated | lysoPC 16:0 | [M+H]+ | 7.69 | Positive |
| lysoPC | 16 | 0 | < 20 | Saturated | lysoPC 16:0 | [M+H]+ | 8.81 | Positive |
| lysoPC | 17 | 0 | < 20 | Saturated | lysoPC 17:0 | [M+H]+ | 6.98 | Positive |
| lysoPC | 17 | 1 | < 20 | Monosaturated | lysoPC 17:1 | [M+H]+ | 10.34 | Positive |
| lysoPC | 19 | 0 | < 20 | Saturated | lysoPC 19:0 | [M+H]+ | 10.56 | Positive |
| lysoPC | 20 | 0 | 20-40 | Saturated | lysoPC 20:0 | [M+H]+ | 9.74 | Positive |
| lysoPC | 20 | 1 | 20-40 | Monosaturated | lysoPC 20:1 | [M+H]+ | 9.69 | Positive |
| lysoPC | 20 | 2 | 20-40 | Polysaturated | lysoPC 20:2 | [M+H]+ | 8.21 | Positive |
| lysoPC | 21 | 0 | 20-40 | Saturated | lysoPC 21:0 | [M+H]+ | 7.81 | Positive |
| lysoPC | 22 | 5 | 20-40 | Polysaturated | lysoPC 22:5 | [M+H]+ | 10.09 | Positive |
| lysoPC | 23 | 0 | 20-40 | Saturated | lysoPC 23:0 | [M+H]+ | 9.30 | Positive |
| lysoPC | 24 | 0 | 20-40 | Saturated | lysoPC 24:0 | [M+H]+ | 10.79 | Positive |
| lysoPC | 26 | 0 | 20-40 | Saturated | lysoPC 26:0 | [M+H]+ | 8.27 | Positive |
| lysoPC | 26 | 1 | 20-40 | Monosaturated | lysoPC 26:1 | [M+H]+ | 7.92 | Positive |
| PA | 34 | 0 | 20-40 | Saturated | PA 34:0 | [M+Na]+ | 9.49 | Positive |
| PC | 28 | 0 | 20-40 | Saturated | PC 28:0 | [M+Na]+ | 7.08 | Positive |
| PC | 30 | 0 | 20-40 | Saturated | PC 30:0 | [M+Na]+ | 7.48 | Positive |
| PC | 30 | 0 | 20-40 | Saturated | PC 30:0 | [M+Na]+ | 8.02 | Positive |
| PC | 30 | 0 | 20-40 | Saturated | PC 30:0 | [M+Na]+ | 10.09 | Positive |
| PC | 30 | 0 | 20-40 | Saturated | PC 30:0 | [M+Na]+ | 9.98 | Positive |
| PC | 30 | 1 | 20-40 | Monosaturated | PC 30:1 | [M+Na]+ | 10.53 | Positive |
| PC | 30 | 1 | 20-40 | Monosaturated | PC 30:1 | [M+Na]+ | 8.90 | Positive |
| PC | 30 | 1 | 20-40 | Monosaturated | PC 30:1 | [M+Na]+ | 7.15 | Positive |
| PC | 31 | 1 | 20-40 | Monosaturated | PC 31:1 | [M+Na]+ | 9.29 | Positive |
| PC | 32 | 0 | 20-40 | Saturated | PC 32:0 | [M+H]+ | 10.38 | Positive |
| PC | 32 | 0 | 20-40 | Saturated | PC 32:0 | [M+Na]+ | 9.34 | Positive |
| PC | 32 | 0 | 20-40 | Saturated | PC 32:0 | [M+Na]+ | 8.49 | Positive |
| PC | 32 | 0 | 20-40 | Saturated | PC 32:0 | [M+Na]+ | 9.89 | Positive |
| PC | 32 | 0 | 20-40 | Saturated | PC 32:0 | [M+Na]+ | 10.60 | Positive |
| PC | 32 | 1 | 20-40 | Monosaturated | PC 32:1 | [M+Na]+ | 10.31 | Positive |
| PC | 32 | 1 | 20-40 | Monosaturated | PC 32:1 | [M+Na]+ | 9.79 | Positive |
| PC | 32 | 2 | 20-40 | Polysaturated | PC 32:2 | [M+Na]+ | 10.23 | Positive |
| PC | 32 | 2 | 20-40 | Polysaturated | PC 32:2 | [M+Na]+ | 9.54 | Positive |
| PC | 32 | 4 | 20-40 | Polysaturated | PC 32:4 | [M+H]+ | 7.22 | Positive |
| PC | 33 | 2 | 20-40 | Polysaturated | PC 33:2 | [M+Na]+ | 6.38 | Positive |
| PC | 33 | 4 | 20-40 | Polysaturated | PC 33:4 | [M+H]+ | 9.78 | Positive |
| PC | 34 | 1 | 20-40 | Monosaturated | PC 34:1 | [M+Na]+ | 10.31 | Positive |
| PC | 34 | 2 | 20-40 | Polysaturated | PC 34:2 | [M+Na]+ | 10.19 | Positive |
| PC | 34 | 2 | 20-40 | Polysaturated | PC 34:2 | [M+Na]+ | 8.24 | Positive |
| PC | 34 | 2 | 20-40 | Polysaturated | PC 34:2 | [M+Na]+ | 7.45 | Positive |
| PC | 34 | 3 | 20-40 | Polysaturated | PC 34:3 | [M+Na]+ | 9.86 | Positive |
| PC | 34 | 3 | 20-40 | Polysaturated | PC 34:3 | [M+Na]+ | 9.68 | Positive |
| PC | 34 | 4 | 20-40 | Polysaturated | PC 34:4 | [M+H]+ | 10.06 | Positive |
| PC | 35 | 1 | 20-40 | Monosaturated | PC 35:1 | [M+Na]+ | 6.32 | Positive |
| PC | 35 | 4 | 20-40 | Polysaturated | PC 35:4 | [M+Na]+ | 1.61 | Positive |
| PC | 35 | 5 | 20-40 | Polysaturated | PC 35:5 | [M+H]+ | 8.24 | Positive |
| PC | 35 | 5 | 20-40 | Polysaturated | PC 35:5 | [M+H]+ | 9.35 | Positive |
| PC | 35 | 5 | 20-40 | Polysaturated | PC 35:5 | [M+H]+ | 9.26 | Positive |
| PC | 35 | 5 | 20-40 | Polysaturated | PC 35:5 | [M+Na]+ | 7.10 | Positive |
| PC | 36 | 2 | 20-40 | Polysaturated | PC 36:2 | [M+Na]+ | 7.43 | Positive |
| PC | 36 | 2 | 20-40 | Polysaturated | PC 36:2 | [M+Na]+ | 5.59 | Positive |
| PC | 36 | 3 | 20-40 | Polysaturated | PC 36:3 | [M+H]+ | 5.20 | Positive |
| PC | 36 | 3 | 20-40 | Polysaturated | PC 36:3 | [M+H]+ | 10.17 | Positive |
| PC | 36 | 3 | 20-40 | Polysaturated | PC 36:3 | [M+Na]+ | 4.52 | Positive |
| PC | 36 | 4 | 20-40 | Polysaturated | PC 36:4 | [M+H]+ | 10.14 | Positive |
| PC | 36 | 4 | 20-40 | Polysaturated | PC 36:4 | [M+H]+ | 10.82 | Positive |
| PC | 36 | 4 | 20-40 | Polysaturated | PC 36:4 | [M+Na]+ | 5.89 | Positive |
| PC | 36 | 4 | 20-40 | Polysaturated | PC 36:4 | [M+Na]+ | 9.55 | Positive |
| PC | 36 | 4 | 20-40 | Polysaturated | PC 36:4 | [M+Na]+ | 10.54 | Positive |
| PC | 36 | 5 | 20-40 | Polysaturated | PC 36:5 | [M+Na]+ | 6.51 | Positive |
| PC | 36 | 5 | 20-40 | Polysaturated | PC 36:5 | [M+Na]+ | 10.14 | Positive |
| PC | 36 | 5 | 20-40 | Polysaturated | PC 36:5 | [M+Na]+ | 9.87 | Positive |
| PC | 37 | 2 | 20-40 | Polysaturated | PC 37:2 | [M+Na]+ | 9.81 | Positive |
| PC | 37 | 5 | 20-40 | Polysaturated | PC 37:5 | [M+H]+ | 3.89 | Positive |
| PC | 37 | 6 | 20-40 | Polysaturated | PC 37:6 | [M+Na]+ | 8.21 | Positive |
| PC | 37 | 6 | 20-40 | Polysaturated | PC 37:6 | [M+Na]+ | 7.15 | Positive |
| PC | 38 | 1 | 20-40 | Monosaturated | PC 38:1 | [M+Na]+ | 10.44 | Positive |
| PC | 38 | 1 | 20-40 | Monosaturated | PC 38:1 | [M+Na]+ | 6.70 | Positive |
| PC | 38 | 1 | 20-40 | Monosaturated | PC 38:1 | [M+Na]+ | 6.16 | Positive |
| PC | 38 | 1 | 20-40 | Monosaturated | PC 38:1 | [M+Na]+ | 5.86 | Positive |
| PC | 38 | 2 | 20-40 | Polysaturated | PC 38:2 | [M+Na]+ | 11.00 | Positive |
| PC | 38 | 3 | 20-40 | Polysaturated | PC 38:3 | [M+H]+ | 9.97 | Positive |
| PC | 38 | 4 | 20-40 | Polysaturated | PC 38:4 | [M+Na]+ | 2.50 | Positive |
| PC | 38 | 4 | 20-40 | Polysaturated | PC 38:4 | [M+Na]+ | 6.88 | Positive |
| PC | 38 | 4 | 20-40 | Polysaturated | PC 38:4 | [M+Na]+ | 5.23 | Positive |
| PC | 38 | 5 | 20-40 | Polysaturated | PC 38:5 | [M+H]+ | 5.64 | Positive |
| PC | 38 | 5 | 20-40 | Polysaturated | PC 38:5 | [M+Na]+ | 11.00 | Positive |
| PC | 38 | 6 | 20-40 | Polysaturated | PC 38:6 | [M+H]+ | 10.48 | Positive |
| PC | 38 | 6 | 20-40 | Polysaturated | PC 38:6 | [M+Na]+ | 9.88 | Positive |
| PC | 38 | 6 | 20-40 | Polysaturated | PC 38:6 | [M+Na]+ | 10.03 | Positive |
| PC | 38 | 7 | 20-40 | Polysaturated | PC 38:6 | [M+Na]+ | 9.64 | Positive |
| PC | 38 | 7 | 20-40 | Polysaturated | PC 38:7 | [M+H]+ | 10.31 | Positive |
| PC | 38 | 7 | 20-40 | Polysaturated | PC 38:7 | [M+H]+ | 6.36 | Positive |
| PC | 38 | 7 | 20-40 | Polysaturated | PC 38:7 | [M+Na]+ | 6.74 | Positive |
| PC | 38 | 7 | 20-40 | Polysaturated | PC 38:7 | [M+Na]+ | 10.14 | Positive |
| PC | 38 | 8 | 20-40 | Polysaturated | PC 38:8 | [M+H]+ | 9.49 | Positive |
| PC | 38 | 8 | 20-40 | Polysaturated | PC 38:8 | [M+Na]+ | 10.24 | Positive |
| PC | 38 | 8 | 20-40 | Polysaturated | PC 38:8 | [M+Na]+ | 6.11 | Positive |
| PC | 38 | 9 | 20-40 | Polysaturated | PC 38:9 | [M+Na]+ | 7.82 | Positive |
| PC | 39 | 5 | 20-40 | Polysaturated | PC 39:5 | [M+H]+ | 6.90 | Positive |
| PC | 39 | 7 | 20-40 | Polysaturated | PC 39:7 | [M+H]+ | 7.58 | Positive |
| PC | 40 | 0 | >= 40 | Saturated | PC 40:0 | [M+Na]+ | 9.83 | Positive |
| PC | 40 | 1 | >= 40 | Monosaturated | PC 40:1 | [M+Na]+ | 9.55 | Positive |
| PC | 40 | 1 | >= 40 | Monosaturated | PC 40:1 | [M+Na]+ | 8.91 | Positive |
| PC | 40 | 1 | >= 40 | Monosaturated | PC 40:1 | [M+Na]+ | 5.60 | Positive |
| PC | 40 | 1 | >= 40 | Monosaturated | PC 40:1 | [M+Na]+ | 10.87 | Positive |
| PC | 40 | 10 | >= 40 | Polysaturated | PC 40:10 | [M+H]+ | 5.78 | Positive |
| PC | 40 | 2 | >= 40 | Polysaturated | PC 40:2 | [M+Na]+ | 9.97 | Positive |
| PC | 40 | 2 | >= 40 | Polysaturated | PC 40:2 | [M+Na]+ | 10.00 | Positive |
| PC | 40 | 3 | >= 40 | Polysaturated | PC 40:3 | [M+Na]+ | 7.32 | Positive |
| PC | 40 | 3 | >= 40 | Polysaturated | PC 40:3 | [M+Na]+ | 10.44 | Positive |
| PC | 40 | 3 | >= 40 | Polysaturated | PC 40:3 | [M+Na]+ | 5.73 | Positive |
| PC | 40 | 4 | >= 40 | Polysaturated | PC 40:4 | [M+H]+ | 5.99 | Positive |
| PC | 40 | 4 | >= 40 | Polysaturated | PC 40:4 | [M+Na]+ | 9.91 | Positive |
| PC | 40 | 4 | >= 40 | Polysaturated | PC 40:4 | [M+Na]+ | 5.36 | Positive |
| PC | 40 | 4 | >= 40 | Polysaturated | PC 40:4 | [M+Na]+ | 6.13 | Positive |
| PC | 40 | 4 | >= 40 | Polysaturated | PC 40:4 | [M+Na]+ | 10.09 | Positive |
| PC | 40 | 4 | >= 40 | Polysaturated | PC 40:4 | [M+Na]+ | 10.31 | Positive |
| PC | 40 | 5 | >= 40 | Polysaturated | PC 40:5 | [M+H]+ | 10.46 | Positive |
| PC | 40 | 5 | >= 40 | Polysaturated | PC 40:5 | [M+H]+ | 9.83 | Positive |
| PC | 40 | 6 | >= 40 | Polysaturated | PC 40:6 | [M+Na]+ | 10.51 | Positive |
| PC | 40 | 7 | >= 40 | Polysaturated | PC 40:7 | [M+H]+ | 9.87 | Positive |
| PC | 40 | 7 | >= 40 | Polysaturated | PC 40:7 | [M+H]+ | 1.74 | Positive |
| PC | 40 | 7 | >= 40 | Polysaturated | PC 40:7 | [M+Na]+ | 9.12 | Positive |
| PC | 40 | 7 | >= 40 | Polysaturated | PC 40:7 | [M+Na]+ | 10.05 | Positive |
| PC | 40 | 8 | >= 40 | Polysaturated | PC 40:8 | [M+H]+ | 6.60 | Positive |
| PC | 40 | 8 | >= 40 | Polysaturated | PC 40:8 | [M+Na]+ | 8.54 | Positive |
| PC | 40 | 9 | >= 40 | Polysaturated | PC 40:9 | [M+H]+ | 5.43 | Positive |
| PC | 42 | 0 | >= 40 | Saturated | PC 42:0 | [M+Na]+ | 4.93 | Positive |
| PC | 42 | 0 | >= 40 | Saturated | PC 42:0 | [M+Na]+ | 7.34 | Positive |
| PC | 42 | 1 | >= 40 | Monosaturated | PC 42:1 | [M+Na]+ | 10.27 | Positive |
| PC | 42 | 1 | >= 40 | Monosaturated | PC 42:1 | [M+Na]+ | 7.64 | Positive |
| PC | 42 | 1 | >= 40 | Monosaturated | PC 42:1 | [M+Na]+ | 10.04 | Positive |
| PC | 42 | 2 | >= 40 | Polysaturated | PC 42:2 | [M+Na]+ | 9.87 | Positive |
| PC | 42 | 3 | >= 40 | Polysaturated | PC 42:3 | [M+Na]+ | 10.51 | Positive |
| PC | 42 | 3 | >= 40 | Polysaturated | PC 42:3 | [M+Na]+ | 7.93 | Positive |
| PC | 42 | 3 | >= 40 | Polysaturated | PC 42:3 | [M+Na]+ | 7.86 | Positive |
| PC | 42 | 4 | >= 40 | Polysaturated | PC 42:4 | [M+Na]+ | 7.52 | Positive |
| PC | 42 | 4 | >= 40 | Polysaturated | PC 42:4 | [M+Na]+ | 10.05 | Positive |
| PC | 42 | 4 | >= 40 | Polysaturated | PC 42:4 | [M+Na]+ | 10.32 | Positive |
| PC | 42 | 4 | >= 40 | Polysaturated | PC 42:4 | [M+Na]+ | 9.97 | Positive |
| PC | 42 | 5 | >= 40 | Polysaturated | PC 42:5 | [M+H]+ | 5.46 | Positive |
| PC | 42 | 5 | >= 40 | Polysaturated | PC 42:5 | [M+Na]+ | 9.90 | Positive |
| PC | 42 | 5 | >= 40 | Polysaturated | PC 42:5 | [M+Na]+ | 6.32 | Positive |
| PC | 42 | 6 | >= 40 | Polysaturated | PC 42:6 | [M+Na]+ | 7.70 | Positive |
| PC | 42 | 6 | >= 40 | Polysaturated | PC 42:6 | [M+Na]+ | 7.32 | Positive |
| PC | 42 | 6 | >= 40 | Polysaturated | PC 42:6 | [M+Na]+ | 6.04 | Positive |
| PC | 42 | 6 | >= 40 | Polysaturated | PC 42:6 | [M+Na]+ | 8.87 | Positive |
| PC | 42 | 9 | >= 40 | Polysaturated | PC 42:9 | [M+Na]+ | 7.20 | Positive |
| PC | 44 | 0 | >= 40 | Saturated | PC 44:0 | [M+Na]+ | 6.67 | Positive |
| PC | 44 | 1 | >= 40 | Monosaturated | PC 44:1 | [M+Na]+ | 7.78 | Positive |
| PC | 44 | 2 | >= 40 | Polysaturated | PC 44:2 | [M+Na]+ | 10.50 | Positive |
| PC | 44 | 3 | >= 40 | Polysaturated | PC 44:3 | [M+Na]+ | 5.62 | Positive |
| PC | 44 | 5 | >= 40 | Polysaturated | PC 44:5 | [M+H]+ | 8.88 | Positive |
| PC | 46 | 1 | >= 40 | Monosaturated | PC 46:1 | [M+Na]+ | 10.77 | Positive |
| PC | 46 | 1 | >= 40 | Monosaturated | PC 46:1 | [M+Na]+ | 7.88 | Positive |
| PC | 46 | 2 | >= 40 | Polysaturated | PC 46:2 | [M+Na]+ | 7.70 | Positive |
| PC | 46 | 2 | >= 40 | Polysaturated | PC 46:2 | [M+Na]+ | 5.60 | Positive |
| PC | 46 | 4 | >= 40 | Polysaturated | PC 46:4 | [M+Na]+ | 9.51 | Positive |
| PC | 46 | 4 | >= 40 | Polysaturated | PC 46:4 | [M+Na]+ | 10.29 | Positive |
| PC | 48 | 5 | >= 40 | Polysaturated | PC 48:5 | [M+Na]+ | 10.79 | Positive |
| PE | 37 | 7 | 20-40 | Polysaturated | PE 37:7 | [M+Na]+ | 6.35 | Positive |
| plasmenyl-PC | 30 | 0 | 20-40 | Saturated | plasmenyl-PC 30:0 | [M+Na]+ | 6.65 | Positive |
| plasmenyl-PC | 32 | 0 | 20-40 | Saturated | plasmenyl-PC 32:0 | [M+Na]+ | 10.55 | Positive |
| plasmenyl-PC | 32 | 1 | 20-40 | Monosaturated | plasmenyl-PC 32:1 | [M+Na]+ | 5.58 | Positive |
| plasmenyl-PC | 33 | 0 | 20-40 | Saturated | plasmenyl-PC 33:0 | [M+Na]+ | 10.08 | Positive |
| plasmenyl-PC | 34 | 0 | 20-40 | Saturated | plasmenyl-PC 34:0 | [M+Na]+ | 6.83 | Positive |
| plasmenyl-PC | 34 | 1 | 20-40 | Monosaturated | plasmenyl-PC 34:1 | [M+Na]+ | 8.76 | Positive |
| plasmenyl-PC | 36 | 0 | 20-40 | Saturated | plasmenyl-PC 36:0 | [M+Na]+ | 5.61 | Positive |
| plasmenyl-PC | 36 | 0 | 20-40 | Saturated | plasmenyl-PC 36:0 | [M+Na]+ | 9.34 | Positive |
| plasmenyl-PC | 37 | 0 | 20-40 | Saturated | plasmenyl-PC 37:0 | [M+Na]+ | 7.04 | Positive |
| plasmenyl-PC | 38 | 0 | 20-40 | Saturated | plasmenyl-PC 38:0 | [M+Na]+ | 10.40 | Positive |
| plasmenyl-PC | 38 | 1 | 20-40 | Monosaturated | plasmenyl-PC 38:1 | [M+Na]+ | 10.66 | Positive |
| plasmenyl-PC | 38 | 1 | 20-40 | Monosaturated | plasmenyl-PC 38:1 | [M+Na]+ | 10.45 | Positive |
| plasmenyl-PC | 38 | 2 | 20-40 | Polysaturated | plasmenyl-PC 38:2 | [M+Na]+ | 10.02 | Positive |
| plasmenyl-PC | 38 | 3 | 20-40 | Polysaturated | plasmenyl-PC 38:3 | [M+Na]+ | 10.03 | Positive |
| plasmenyl-PC | 38 | 5 | 20-40 | Polysaturated | plasmenyl-PC 38:5 | [M+Na]+ | 6.65 | Positive |
| plasmenyl-PC | 39 | 0 | 20-40 | Saturated | plasmenyl-PC 39:0 | [M+Na]+ | 9.93 | Positive |
| plasmenyl-PC | 40 | 3 | >= 40 | Polysaturated | plasmenyl-PC 40:3 | [M+Na]+ | 10.38 | Positive |
| plasmenyl-PC | 40 | 4 | >= 40 | Polysaturated | plasmenyl-PC 40:4 | [M+Na]+ | 6.33 | Positive |
| plasmenyl-PC | 40 | 4 | >= 40 | Polysaturated | plasmenyl-PC 40:4 | [M+Na]+ | 6.82 | Positive |
| plasmenyl-PC | 40 | 4 | >= 40 | Polysaturated | plasmenyl-PC 40:4 | [M+Na]+ | 5.31 | Positive |
| plasmenyl-PC | 40 | 6 | >= 40 | Polysaturated | plasmenyl-PC 40:6 | [M+Na]+ | 6.26 | Positive |
| plasmenyl-PC | 42 | 4 | >= 40 | Polysaturated | plasmenyl-PC 42:4 | [M+Na]+ | 5.62 | Positive |
| plasmenyl-PC | 43 | 0 | >= 40 | Saturated | plasmenyl-PC 43:0 | [M+Na]+ | 4.40 | Positive |
| plasmenyl-PE | 34 | 1 | 20-40 | Monosaturated | plasmenyl-PE 34:1 | [M+Na]+ | 5.63 | Positive |
| plasmenyl-PE | 34 | 2 | 20-40 | Polysaturated | plasmenyl-PE 34:2 | [M+Na]+ | 6.04 | Positive |
| plasmenyl-PE | 36 | 1 | 20-40 | Monosaturated | plasmenyl-PE 36:1 | [M+H]+ | 9.80 | Positive |
| plasmenyl-PE | 36 | 1 | 20-40 | Monosaturated | plasmenyl-PE 36:1 | [M+Na]+ | 7.33 | Positive |
| plasmenyl-PE | 36 | 3 | 20-40 | Polysaturated | plasmenyl-PE 36:3 | [M+H]+ | 9.15 | Positive |
| plasmenyl-PE | 36 | 4 | 20-40 | Polysaturated | plasmenyl-PE 36:4 | [M+H]+ | 10.24 | Positive |
| plasmenyl-PE | 36 | 5 | 20-40 | Polysaturated | plasmenyl-PE 36:5 | [M+H]+ | 9.84 | Positive |
| plasmenyl-PE | 38 | 1 | 20-40 | Monosaturated | plasmenyl-PE 38:1 | [M+Na]+ | 5.25 | Positive |
| plasmenyl-PE | 38 | 3 | 20-40 | Polysaturated | plasmenyl-PE 38:3 | [M+H]+ | 10.77 | Positive |
| plasmenyl-PE | 38 | 4 | 20-40 | Polysaturated | plasmenyl-PE 38:4 | [M+H]+ | 8.83 | Positive |
| plasmenyl-PE | 38 | 4 | 20-40 | Polysaturated | plasmenyl-PE 38:4 | [M+H]+ | 9.24 | Positive |
| plasmenyl-PE | 38 | 5 | 20-40 | Polysaturated | plasmenyl-PE 38:5 | [M+H]+ | 9.40 | Positive |
| plasmenyl-PE | 40 | 2 | >= 40 | Polysaturated | plasmenyl-PE 40:2 | [M+Na]+ | 9.49 | Positive |
| plasmenyl-PE | 42 | 4 | >= 40 | Polysaturated | plasmenyl-PE 42:4 | [M+Na]+ | 6.34 | Positive |
| SM | 34 | 0 | 20-40 | Saturated | SM 34:0 | [M+Na]+ | 10.66 | Positive |
| SM | 34 | 1 | 20-40 | Monosaturated | SM 34:1 | [M+Na]+ | 10.26 | Positive |
| SM | 40 | 1 | >= 40 | Monosaturated | SM 40:1 | [M+Na]+ | 7.45 | Positive |
| SM | 42 | 2 | >= 40 | Polysaturated | SM 42:2 | [M+Na]+ | 9.58 | Positive |
| SM | 42 | 4 | >= 40 | Polysaturated | SM 42:4 | [M]+ | 5.91 | Positive |
| SM | 43 | 4 | >= 40 | Polysaturated | SM 43:4 | [M]+ | 5.09 | Positive |
| SM | 43 | 5 | >= 40 | Polysaturated | SM 43:5 | [M]+ | 7.82 | Positive |
| SM | 44 | 1 | >= 40 | Monosaturated | SM 44:1 | [M+Na]+ | 6.65 | Positive |
| TG | 40 | 0 | >= 40 | Saturated | TG 40:0 | [M+NH4]+ | 7.39 | Positive |
| TG | 42 | 0 | >= 40 | Saturated | TG 42:0 | [M+NH4]+ | 9.94 | Positive |
| TG | 42 | 1 | >= 40 | Monosaturated | TG 42:1 | [M+NH4]+ | 3.45 | Positive |
| TG | 42 | 1 | >= 40 | Monosaturated | TG 42:1 | [M+NH4]+ | 5.95 | Positive |
| TG | 44 | 1 | >= 40 | Monosaturated | TG 44:1 | [M+NH4]+ | 8.24 | Positive |
| TG | 44 | 1 | >= 40 | Monosaturated | TG 44:1 | [M+NH4]+ | 5.61 | Positive |
| TG | 44 | 2 | >= 40 | Polysaturated | TG 44:2 | [M+NH4]+ | 8.75 | Positive |
| TG | 44 | 2 | >= 40 | Polysaturated | TG 44:2 | [M+NH4]+ | 5.62 | Positive |
| TG | 46 | 0 | >= 40 | Saturated | TG 46:0 | [M+Na]+ | 1.91 | Positive |
| TG | 46 | 0 | >= 40 | Saturated | TG 46:0 | [M+NH4]+ | 7.04 | Positive |
| TG | 46 | 0 | >= 40 | Saturated | TG 46:0 | [M+NH4]+ | 5.62 | Positive |
| TG | 46 | 1 | >= 40 | Monosaturated | TG 46:1 | [M+Na]+ | 8.41 | Positive |
| TG | 46 | 1 | >= 40 | Monosaturated | TG 46:1 | [M+NH4]+ | 5.80 | Positive |
| TG | 46 | 2 | >= 40 | Polysaturated | TG 46:2 | [M+Na]+ | 6.26 | Positive |
| TG | 46 | 2 | >= 40 | Polysaturated | TG 46:2 | [M+NH4]+ | 6.92 | Positive |
| TG | 48 | 0 | >= 40 | Saturated | TG 48:0 | [M+Na]+ | 7.01 | Positive |
| TG | 48 | 0 | >= 40 | Saturated | TG 48:0 | [M+NH4]+ | 5.60 | Positive |
| TG | 48 | 1 | >= 40 | Monosaturated | TG 48:1 | [M+Na]+ | 8.02 | Positive |
| TG | 48 | 1 | >= 40 | Monosaturated | TG 48:1 | [M+NH4]+ | 9.87 | Positive |
| TG | 48 | 2 | >= 40 | Polysaturated | TG 48:2 | [M+Na]+ | 7.33 | Positive |
| TG | 48 | 2 | >= 40 | Polysaturated | TG 48:2 | [M+Na]+ | 4.91 | Positive |
| TG | 48 | 2 | >= 40 | Polysaturated | TG 48:2 | [M+NH4]+ | 6.97 | Positive |
| TG | 48 | 2 | >= 40 | Polysaturated | TG 48:2 | [M+NH4]+ | 5.61 | Positive |
| TG | 48 | 3 | >= 40 | Polysaturated | TG 48:3 | [M+Na]+ | 7.00 | Positive |
| TG | 48 | 3 | >= 40 | Polysaturated | TG 48:3 | [M+NH4]+ | 3.04 | Positive |
| TG | 49 | 2 | >= 40 | Polysaturated | TG 49:2 | [M+Na]+ | 6.62 | Positive |
| TG | 49 | 2 | >= 40 | Polysaturated | TG 49:2 | [M+NH4]+ | 9.08 | Positive |
| TG | 50 | 0 | >= 40 | Saturated | TG 50:0 | [M+NH4]+ | 9.83 | Positive |
| TG | 50 | 0 | >= 40 | Saturated | TG 50:0 | [M+NH4]+ | 6.25 | Positive |
| TG | 50 | 1 | >= 40 | Monosaturated | TG 50:1 | [M+Na]+ | 6.54 | Positive |
| TG | 50 | 1 | >= 40 | Monosaturated | TG 50:1 | [M+NH4]+ | 5.17 | Positive |
| TG | 50 | 1 | >= 40 | Monosaturated | TG 50:1 | [M+NH4]+ | 7.66 | Positive |
| TG | 50 | 2 | >= 40 | Polysaturated | TG 50:2 | [M+NH4]+ | 7.91 | Positive |
| TG | 50 | 2 | >= 40 | Polysaturated | TG 50:2 | [M+NH4]+ | 10.82 | Positive |
| TG | 50 | 3 | >= 40 | Polysaturated | TG 50:3 | [M+Na]+ | 9.18 | Positive |
| TG | 50 | 3 | >= 40 | Polysaturated | TG 50:3 | [M+Na]+ | 10.03 | Positive |
| TG | 50 | 3 | >= 40 | Polysaturated | TG 50:3 | [M+NH4]+ | 11.18 | Positive |
| TG | 50 | 3 | >= 40 | Polysaturated | TG 50:3 | [M+NH4]+ | 5.85 | Positive |
| TG | 50 | 4 | >= 40 | Polysaturated | TG 50:4 | [M+Na]+ | 9.57 | Positive |
| TG | 50 | 4 | >= 40 | Polysaturated | TG 50:4 | [M+NH4]+ | 7.03 | Positive |
| TG | 50 | 4 | >= 40 | Polysaturated | TG 50:4 | [M+NH4]+ | 6.00 | Positive |
| TG | 50 | 5 | >= 40 | Polysaturated | TG 50:5 | [M+NH4]+ | 9.58 | Positive |
| TG | 51 | 1 | >= 40 | Monosaturated | TG 51:1 | [M+Na]+ | 10.44 | Positive |
| TG | 51 | 1 | >= 40 | Monosaturated | TG 51:1 | [M+NH4]+ | 10.78 | Positive |
| TG | 51 | 1 | >= 40 | Monosaturated | TG 51:1 | [M+NH4]+ | 6.62 | Positive |
| TG | 51 | 2 | >= 40 | Polysaturated | TG 51:2 | [M+Na]+ | 8.92 | Positive |
| TG | 51 | 2 | >= 40 | Polysaturated | TG 51:2 | [M+NH4]+ | 6.32 | Positive |
| TG | 51 | 2 | >= 40 | Polysaturated | TG 51:2 | [M+NH4]+ | 6.56 | Positive |
| TG | 51 | 3 | >= 40 | Polysaturated | TG 51:3 | [M+Na]+ | 10.03 | Positive |
| TG | 51 | 4 | >= 40 | Polysaturated | TG 51:4 | [M+NH4]+ | 8.02 | Positive |
| TG | 52 | 0 | >= 40 | Saturated | TG 52:0 | [M+NH4]+ | 10.40 | Positive |
| TG | 52 | 0 | >= 40 | Saturated | TG 52:0 | [M+NH4]+ | 10.43 | Positive |
| TG | 52 | 1 | >= 40 | Monosaturated | TG 52:1 | [M+Na]+ | 5.59 | Positive |
| TG | 52 | 1 | >= 40 | Monosaturated | TG 52:1 | [M+NH4]+ | 6.91 | Positive |
| TG | 52 | 2 | >= 40 | Polysaturated | TG 52:2 | [M+Na]+ | 7.48 | Positive |
| TG | 52 | 2 | >= 40 | Polysaturated | TG 52:2 | [M+NH4]+ | 9.80 | Positive |
| TG | 52 | 3 | >= 40 | Polysaturated | TG 52:3 | [M+Na]+ | 9.82 | Positive |
| TG | 52 | 3 | >= 40 | Polysaturated | TG 52:3 | [M+NH4]+ | 6.65 | Positive |
| TG | 52 | 3 | >= 40 | Polysaturated | TG 52:3 | [M+NH4]+ | 11.26 | Positive |
| TG | 52 | 4 | >= 40 | Polysaturated | TG 52:4 | [M+Na]+ | 10.42 | Positive |
| TG | 52 | 4 | >= 40 | Polysaturated | TG 52:4 | [M+Na]+ | 6.06 | Positive |
| TG | 52 | 4 | >= 40 | Polysaturated | TG 52:4 | [M+Na]+ | 7.49 | Positive |
| TG | 52 | 4 | >= 40 | Polysaturated | TG 52:4 | [M+Na]+ | 5.61 | Positive |
| TG | 52 | 4 | >= 40 | Polysaturated | TG 52:4 | [M+NH4]+ | 4.12 | Positive |
| TG | 52 | 4 | >= 40 | Polysaturated | TG 52:4 | [M+NH4]+ | 5.40 | Positive |
| TG | 52 | 4 | >= 40 | Polysaturated | TG 52:4 | [M+NH4]+ | 10.26 | Positive |
| TG | 52 | 4 | >= 40 | Polysaturated | TG 52:4 | [M+NH4]+ | 5.32 | Positive |
| TG | 52 | 5 | >= 40 | Polysaturated | TG 52:5 | [M+Na]+ | 6.25 | Positive |
| TG | 52 | 5 | >= 40 | Polysaturated | TG 52:5 | [M+Na]+ | 10.12 | Positive |
| TG | 52 | 5 | >= 40 | Polysaturated | TG 52:5 | [M+Na]+ | 7.58 | Positive |
| TG | 52 | 5 | >= 40 | Polysaturated | TG 52:5 | [M+Na]+ | 9.74 | Positive |
| TG | 52 | 5 | >= 40 | Polysaturated | TG 52:5 | [M+NH4]+ | 6.58 | Positive |
| TG | 52 | 5 | >= 40 | Polysaturated | TG 52:5 | [M+NH4]+ | 9.96 | Positive |
| TG | 52 | 6 | >= 40 | Polysaturated | TG 52:6 | [M+Na]+ | 5.89 | Positive |
| TG | 52 | 6 | >= 40 | Polysaturated | TG 52:6 | [M+NH4]+ | 10.24 | Positive |
| TG | 52 | 0 | >= 40 | Saturated | TG 52:6 | [M+NH4]+ | 5.82 | Positive |
| TG | 53 | 0 | >= 40 | Saturated | TG 53:0 | [M+Na]+ | 7.33 | Positive |
| TG | 53 | 1 | >= 40 | Monosaturated | TG 53:0 | [M+NH4]+ | 8.56 | Positive |
| TG | 53 | 1 | >= 40 | Monosaturated | TG 53:1 | [M+Na]+ | 9.55 | Positive |
| TG | 53 | 2 | >= 40 | Polysaturated | TG 53:1 | [M+NH4]+ | 9.68 | Positive |
| TG | 53 | 2 | >= 40 | Polysaturated | TG 53:2 | [M+Na]+ | 5.61 | Positive |
| TG | 53 | 2 | >= 40 | Polysaturated | TG 53:2 | [M+NH4]+ | 6.28 | Positive |
| TG | 53 | 2 | >= 40 | Polysaturated | TG 53:2 | [M+NH4]+ | 10.34 | Positive |
| TG | 53 | 3 | >= 40 | Polysaturated | TG 53:3 | [M+NH4]+ | 5.62 | Positive |
| TG | 53 | 4 | >= 40 | Polysaturated | TG 53:4 | [M+Na]+ | 1.79 | Positive |
| TG | 53 | 4 | >= 40 | Polysaturated | TG 53:4 | [M+NH4]+ | 6.71 | Positive |
| TG | 53 | 4 | >= 40 | Polysaturated | TG 53:4 | [M+NH4]+ | 11.01 | Positive |
| TG | 53 | 5 | >= 40 | Polysaturated | TG 53:5 | [M+Na]+ | 6.22 | Positive |
| TG | 53 | 5 | >= 40 | Polysaturated | TG 53:5 | [M+Na]+ | 5.51 | Positive |
| TG | 53 | 5 | >= 40 | Polysaturated | TG 53:5 | [M+NH4]+ | 6.19 | Positive |
| TG | 54 | 1 | >= 40 | Monosaturated | TG 54:1 | [M+Na]+ | 9.75 | Positive |
| TG | 54 | 1 | >= 40 | Monosaturated | TG 54:1 | [M+NH4]+ | 9.68 | Positive |
| TG | 54 | 2 | >= 40 | Polysaturated | TG 54:2 | [M+Na]+ | 10.20 | Positive |
| TG | 54 | 2 | >= 40 | Polysaturated | TG 54:2 | [M+Na]+ | 6.05 | Positive |
| TG | 54 | 2 | >= 40 | Polysaturated | TG 54:2 | [M+NH4]+ | 10.48 | Positive |
| TG | 54 | 2 | >= 40 | Polysaturated | TG 54:2 | [M+NH4]+ | 7.51 | Positive |
| TG | 54 | 3 | >= 40 | Polysaturated | TG 54:3 | [M+NH4]+ | 8.81 | Positive |
| TG | 54 | 4 | >= 40 | Polysaturated | TG 54:4 | [M+NH4]+ | 10.41 | Positive |
| TG | 54 | 5 | >= 40 | Polysaturated | TG 54:5 | [M+Na]+ | 4.95 | Positive |
| TG | 54 | 5 | >= 40 | Polysaturated | TG 54:5 | [M+Na]+ | 10.53 | Positive |
| TG | 54 | 5 | >= 40 | Polysaturated | TG 54:5 | [M+NH4]+ | 10.79 | Positive |
| TG | 54 | 5 | >= 40 | Polysaturated | TG 54:5 | [M+NH4]+ | 10.08 | Positive |
| TG | 54 | 5 | >= 40 | Polysaturated | TG 54:5 | [M+NH4]+ | 6.98 | Positive |
| TG | 54 | 6 | >= 40 | Polysaturated | TG 54:6 | [M+Na]+ | 7.59 | Positive |
| TG | 54 | 6 | >= 40 | Polysaturated | TG 54:6 | [M+Na]+ | 9.95 | Positive |
| TG | 54 | 7 | >= 40 | Polysaturated | TG 54:6 | [M+NH4]+ | 8.23 | Positive |
| TG | 54 | 7 | >= 40 | Polysaturated | TG 54:7 | [M+Na]+ | 6.74 | Positive |
| TG | 54 | 7 | >= 40 | Polysaturated | TG 54:7 | [M+Na]+ | 9.68 | Positive |
| TG | 54 | 7 | >= 40 | Polysaturated | TG 54:7 | [M+NH4]+ | 10.00 | Positive |
| TG | 54 | 7 | >= 40 | Polysaturated | TG 54:7 | [M+NH4]+ | 6.03 | Positive |
| TG | 54 | 8 | >= 40 | Polysaturated | TG 54:8 | [M+Na]+ | 6.79 | Positive |
| TG | 54 | 8 | >= 40 | Polysaturated | TG 54:8 | [M+NH4]+ | 6.63 | Positive |
| TG | 55 | 2 | >= 40 | Polysaturated | TG 55:2 | [M+NH4]+ | 10.68 | Positive |
| TG | 56 | 1 | >= 40 | Monosaturated | TG 56:1 | [M+Na]+ | 10.44 | Positive |
| TG | 56 | 1 | >= 40 | Monosaturated | TG 56:1 | [M+NH4]+ | 5.60 | Positive |
| TG | 56 | 1 | >= 40 | Monosaturated | TG 56:1 | [M+NH4]+ | 8.30 | Positive |
| TG | 56 | 2 | >= 40 | Polysaturated | TG 56:2 | [M+Na]+ | 6.23 | Positive |
| TG | 56 | 2 | >= 40 | Polysaturated | TG 56:2 | [M+NH4]+ | 6.37 | Positive |
| TG | 56 | 3 | >= 40 | Polysaturated | TG 56:3 | [M+NH4]+ | 9.56 | Positive |
| TG | 56 | 3 | >= 40 | Polysaturated | TG 56:3 | [M+NH4]+ | 6.57 | Positive |
| TG | 56 | 4 | >= 40 | Polysaturated | TG 56:4 | [M+Na]+ | 10.58 | Positive |
| TG | 56 | 4 | >= 40 | Polysaturated | TG 56:4 | [M+Na]+ | 6.27 | Positive |
| TG | 56 | 4 | >= 40 | Polysaturated | TG 56:4 | [M+NH4]+ | 9.68 | Positive |
| TG | 56 | 4 | >= 40 | Polysaturated | TG 56:4 | [M+NH4]+ | 5.60 | Positive |
| TG | 56 | 4 | >= 40 | Polysaturated | TG 56:4 | [M+NH4]+ | 8.04 | Positive |
| TG | 56 | 4 | >= 40 | Polysaturated | TG 56:4 | [M+NH4]+ | 7.82 | Positive |
| TG | 56 | 5 | >= 40 | Polysaturated | TG 56:5 | [M+Na]+ | 6.80 | Positive |
| TG | 56 | 5 | >= 40 | Polysaturated | TG 56:5 | [M+NH4]+ | 7.53 | Positive |
| TG | 56 | 5 | >= 40 | Polysaturated | TG 56:5 | [M+NH4]+ | 10.27 | Positive |
| TG | 56 | 5 | >= 40 | Polysaturated | TG 56:5 | [M+NH4]+ | 7.04 | Positive |
| TG | 56 | 6 | >= 40 | Polysaturated | TG 56:6 | [M+Na]+ | 8.85 | Positive |
| TG | 56 | 6 | >= 40 | Polysaturated | TG 56:6 | [M+Na]+ | 10.46 | Positive |
| TG | 56 | 6 | >= 40 | Polysaturated | TG 56:6 | [M+NH4]+ | 9.34 | Positive |
| TG | 56 | 7 | >= 40 | Polysaturated | TG 56:7 | [M+Na]+ | 7.17 | Positive |
| TG | 56 | 7 | >= 40 | Polysaturated | TG 56:7 | [M+Na]+ | 4.81 | Positive |
| TG | 56 | 7 | >= 40 | Polysaturated | TG 56:7 | [M+Na]+ | 10.00 | Positive |
| TG | 56 | 7 | >= 40 | Polysaturated | TG 56:7 | [M+NH4]+ | 6.26 | Positive |
| TG | 56 | 8 | >= 40 | Polysaturated | TG 56:8 | [M+Na]+ | 5.19 | Positive |
| TG | 56 | 8 | >= 40 | Polysaturated | TG 56:8 | [M+Na]+ | 6.91 | Positive |
| TG | 56 | 8 | >= 40 | Polysaturated | TG 56:8 | [M+NH4]+ | 9.20 | Positive |
| TG | 57 | 5 | >= 40 | Polysaturated | TG 57:5 | [M+NH4]+ | 7.45 | Positive |
| TG | 58 | 1 | >= 40 | Monosaturated | TG 58:1 | [M+NH4]+ | 10.87 | Positive |
| TG | 58 | 2 | >= 40 | Polysaturated | TG 58:2 | [M+Na]+ | 9.99 | Positive |
| TG | 58 | 2 | >= 40 | Polysaturated | TG 58:2 | [M+NH4]+ | 10.47 | Positive |
| TG | 58 | 3 | >= 40 | Polysaturated | TG 58:3 | [M+NH4]+ | 7.31 | Positive |
| TG | 58 | 3 | >= 40 | Polysaturated | TG 58:3 | [M+NH4]+ | 6.81 | Positive |
| TG | 58 | 4 | >= 40 | Polysaturated | TG 58:4 | [M+NH4]+ | 5.95 | Positive |
| TG | 58 | 4 | >= 40 | Polysaturated | TG 58:4 | [M+NH4]+ | 8.38 | Positive |
| TG | 58 | 5 | >= 40 | Polysaturated | TG 58:5 | [M+Na]+ | 7.64 | Positive |
| TG | 58 | 5 | >= 40 | Polysaturated | TG 58:5 | [M+NH4]+ | 5.73 | Positive |
| TG | 58 | 5 | >= 40 | Polysaturated | TG 58:5 | [M+NH4]+ | 10.06 | Positive |
| TG | 58 | 6 | >= 40 | Polysaturated | TG 58:6 | [M+Na]+ | 4.94 | Positive |
| TG | 58 | 6 | >= 40 | Polysaturated | TG 58:6 | [M+NH4]+ | 7.59 | Positive |
| TG | 58 | 7 | >= 40 | Polysaturated | TG 58:7 | [M+Na]+ | 7.83 | Positive |
| TG | 58 | 7 | >= 40 | Polysaturated | TG 58:7 | [M+NH4]+ | 7.02 | Positive |
| TG | 58 | 7 | >= 40 | Polysaturated | TG 58:7 | [M+NH4]+ | 7.26 | Positive |
| TG | 58 | 8 | >= 40 | Polysaturated | TG 58:8 | [M+Li]+ | 8.61 | Positive |
| TG | 58 | 8 | >= 40 | Polysaturated | TG 58:8 | [M+Na]+ | 10.64 | Positive |
| TG | 58 | 8 | >= 40 | Polysaturated | TG 58:8 | [M+NH4]+ | 4.94 | Positive |
| TG | 58 | 8 | >= 40 | Polysaturated | TG 58:8 | [M+NH4]+ | 6.32 | Positive |
| TG | 58 | 9 | >= 40 | Polysaturated | TG 58:9 | [M+NH4]+ | 10.99 | Positive |
| TG | 60 | 6 | >= 40 | Polysaturated | TG 60:6 | [M+Na]+ | 7.34 | Positive |
| TG | 60 | 6 | >= 40 | Polysaturated | TG 60:6 | [M+NH4]+ | 2.23 | Positive |
| TG | 60 | 7 | >= 40 | Polysaturated | TG 60:7 | [M+NH4]+ | 9.46 | Positive |
| TG | 60 | 8 | >= 40 | Polysaturated | TG 60:8 | [M+NH4]+ | 5.61 | Positive |
| TG | 60 | 9 | >= 40 | Polysaturated | TG 60:9 | [M+Li]+ | 9.64 | Positive |
| TG | 60 | 9 | >= 40 | Polysaturated | TG 60:9 | [M+NH4]+ | 1.45 | Positive |
| MG | 17 | 0 | < 20 | Saturated | IS MG 17:0; | [M-H]- | 2.87 | Negative |
| PE | 34 | 0 | 20-40 | Saturated | IS PE 34:0 | [M-H]- | 7.31 | Negative |
| PG | 34 | 0 | 20-40 | Saturated | IS PG 34:0 | [M-H]- | 6.31 | Negative |
| CE | 18 | 1 | < 20 | Monosaturated | N-(15Z-tetracosenoyl)-sphing-4-enine | [M-H]- | 6.812 | Negative |
| CE | 18 | 1 | < 20 | Monosaturated | N-(hexadecanoyl)-sphing-4-enine | [M-H]- | 5.4263 | Negative |
| CE | 18 | 1 | < 20 | Monosaturated | N-(octadecanoyl)-sphing-4-enine | [M-H]- | 5.4854 | Negative |
| CE | 18 | 1 | < 20 | Monosaturated | N-(tetracosanoyl)-sphing-4-enine | [M-H]- | 5.6346 | Negative |
| CL | 60 | 0 | >= 40 | Saturated | CL 60:0 | [M-2H](2-) | 6.6772 | Negative |
| CL | 66 | 2 | >= 40 | Polysaturated | CL 66:2 | [M-2H](2-) | 6.5309 | Negative |
| CL | 68 | 1 | >= 40 | Monosaturated | CL 68:1 | [M-2H](2-) | 5.4203 | Negative |
| CL | 68 | 2 | >= 40 | Polysaturated | CL 68:2 | [M-2H](2-) | 5.4812 | Negative |
| CL | 68 | 2 | >= 40 | Polysaturated | CL 68:2 | [M-2H](2-) | 6.095 | Negative |
| CL | 68 | 3 | >= 40 | Polysaturated | CL 68:3 | [M-2H](2-) | 6.1814 | Negative |
| CL | 68 | 4 | >= 40 | Polysaturated | CL 68:4 | [M-2H](2-) | 5.4574 | Negative |
| CL | 68 | 4 | >= 40 | Polysaturated | CL 68:4 | [M-2H](2-) | 6.033 | Negative |
| CL | 68 | 4 | >= 40 | Polysaturated | CL 68:4 | [M-2H](2-) | 6.6875 | Negative |
| CL | 70 | 5 | >= 40 | Polysaturated | CL 70:5 | [M-2H](2-) | 7.1917 | Negative |
| CL | 70 | 5 | >= 40 | Polysaturated | CL 70:5 | [M-2H](2-) | 7.2719 | Negative |
| CL | 70 | 5 | >= 40 | Polysaturated | CL 70:5 | [M-2H](2-) | 6.1668 | Negative |
| CL | 70 | 5 | >= 40 | Polysaturated | CL 70:5 | [M-2H](2-) | 6.6246 | Negative |
| CL | 70 | 6 | >= 40 | Polysaturated | CL 70:6 | [M-2H](2-) | 6.092 | Negative |
| CL | 72 | 0 | >= 40 | Saturated | CL 72:0 | [M-2H](2-) | 6.441 | Negative |
| CL | 72 | 0 | >= 40 | Saturated | CL 72:0 | [M-2H](2-) | 5.7209 | Negative |
| CL | 72 | 0 | >= 40 | Saturated | CL 72:0 | [M-2H](2-) | 6.1464 | Negative |
| CL | 72 | 1 | >= 40 | Monosaturated | CL 72:1 | [M-2H](2-) | 6.0303 | Negative |
| CL | 72 | 3 | >= 40 | Polysaturated | CL 72:3 | [M-2H](2-) | 6.1031 | Negative |
| CL | 72 | 6 | >= 40 | Polysaturated | CL 72:6 | [M-2H](2-) | 6.6902 | Negative |
| CL | 74 | 8 | >= 40 | Polysaturated | CL 74:8 | [M-2H](2-) | 5.2821 | Negative |
| CL | 74 | 8 | >= 40 | Polysaturated | CL 74:8 | [M-2H](2-) | 6.1577 | Negative |
| DGDG | 18 | 0 | < 20 | Saturated | DGDG 18:0 | [M-H]- | 7.4754 | Negative |
| PA | 28 | 0 | 20-40 | Saturated | PA 28:0 | [M-H]- | 5.2983 | Negative |
| PA | 32 | 0 | 20-40 | Saturated | PA 32:0 | [M-H]- | 7.3996 | Negative |
| PA | 34 | 0 | 20-40 | Saturated | PA 34:0 | [M-H]- | 5.9812 | Negative |
| PA | 34 | 1 | 20-40 | Monosaturated | PA 34:1 | [M-H]- | 5.4266 | Negative |
| PA | 34 | 2 | 20-40 | Polysaturated | PA 34:2 | [M-H]- | 5.5224 | Negative |
| PA | 35 | 2 | 20-40 | Polysaturated | PA 35:2 | [M-H]- | 5.334 | Negative |
| PA | 36 | 1 | 20-40 | Monosaturated | PA 36:1 | [M-H]- | 5.5955 | Negative |
| PA | 36 | 2 | 20-40 | Polysaturated | PA 36:2 | [M-H]- | 6.0581 | Negative |
| PA | 36 | 4 | 20-40 | Polysaturated | PA 36:4 | [M-H]- | 6.376 | Negative |
| PA | 38 | 0 | 20-40 | Saturated | PA 38:0 | [M-H]- | 5.842 | Negative |
| PA | 38 | 2 | 20-40 | Polysaturated | PA 38:2 | [M-H]- | 5.7965 | Negative |
| PA | 40 | 4 | >= 40 | Polysaturated | PA 38:4 | [M-H]- | 5.6488 | Negative |
| PA | 40 | 6 | >= 40 | Polysaturated | PA 40:6 | [M-H]- | 5.4253 | Negative |
| PC | 29 | 0 | 20-40 | Saturated | PC 10:0 | [M+HCOO]- | 5.9339 | Negative |
| PC | 30 | 0 | 20-40 | Saturated | PC 29:0 | [M-Ac-H]- | 5.754 | Negative |
| PC | 30 | 0 | 20-40 | Saturated | PC 30:0 | [M-Ac-H]- | 5.4367 | Negative |
| PC | 30 | 0 | 20-40 | Saturated | PC 30:0 | [M-CH3]- | 6.838 | Negative |
| PC | 31 | 0 | 20-40 | Saturated | PC 31:0 | [M-CH3]- | 5.7348 | Negative |
| PC | 32 | 1 | 20-40 | Monosaturated | PC 32:1 | [M-CH3]- | 6.222 | Negative |
| PC | 32 | 1 | 20-40 | Monosaturated | PC 32:1 | [M-CH3]- | 6.8767 | Negative |
| PC | 32 | 1 | 20-40 | Monosaturated | PC 32:1 | [M-CH3]- | 5.9342 | Negative |
| PC | 32 | 2 | 20-40 | Polysaturated | PC 32:2 | [M-Ac-H]- | 5.4125 | Negative |
| PC | 33 | 0 | 20-40 | Saturated | PC 33:0 | [M-Ac-H]- | 5.7449 | Negative |
| PC | 34 | 0 | 20-40 | Saturated | PC 34:0 | [M-Ac-H]- | 5.7828 | Negative |
| PC | 34 | 1 | 20-40 | Monosaturated | PC 34:1 | [M+HCOO]- | 6.4947 | Negative |
| PC | 34 | 1 | 20-40 | Monosaturated | PC 34:1 | [M-Ac-H]- | 5.313 | Negative |
| PC | 34 | 1 | 20-40 | Monosaturated | PC 34:1 | [M-CH3]- | 6.8214 | Negative |
| PC | 34 | 2 | 20-40 | Polysaturated | PC 34:2 | [M+HCOO]- | 5.9695 | Negative |
| PC | 34 | 2 | 20-40 | Polysaturated | PC 34:2 | [M+HCOO]- | 5.301 | Negative |
| PC | 34 | 2 | 20-40 | Polysaturated | PC 34:2 | [M-Ac-H]- | 7.2522 | Negative |
| PC | 34 | 2 | 20-40 | Polysaturated | PC 34:2 | [M-Ac-H]- | 8.9173 | Negative |
| PC | 34 | 2 | 20-40 | Polysaturated | PC 34:2 | [M-CH3]- | 5.5716 | Negative |
| PC | 34 | 2 | 20-40 | Polysaturated | PC 34:2 | [M-CH3]- | 7.2822 | Negative |
| PC | 34 | 3 | 20-40 | Polysaturated | PC 34:3 | [M-Ac-H]- | 6.1824 | Negative |
| PC | 34 | 4 | 20-40 | Polysaturated | PC 34:4 | [M-Ac-H]- | 7.4334 | Negative |
| PC | 35 | 0 | 20-40 | Saturated | PC 35:0 | [M-Ac-H]- | 6.0297 | Negative |
| PC | 35 | 1 | 20-40 | Monosaturated | PC 35:1 | [M-Ac-H]- | 6.438 | Negative |
| PC | 36 | 1 | 20-40 | Monosaturated | PC 36:1 | [M-CH3]- | 6.4655 | Negative |
| PC | 36 | 3 | 20-40 | Polysaturated | PC 36:3 | [M+HCOO]- | 6.814 | Negative |
| PC | 36 | 3 | 20-40 | Polysaturated | PC 36:3 | [M-Ac-H]- | 8.8478 | Negative |
| PC | 36 | 3 | 20-40 | Polysaturated | PC 36:3 | [M-CH3]- | 4.542 | Negative |
| PC | 36 | 4 | 20-40 | Polysaturated | PC 36:4 | [M+HCOO]- | 7.2867 | Negative |
| PC | 36 | 4 | 20-40 | Polysaturated | PC 36:4 | [M-Ac-H]- | 7.3938 | Negative |
| PC | 36 | 4 | 20-40 | Polysaturated | PC 36:4 | [M-CH3]- | 8.8988 | Negative |
| PC | 36 | 5 | 20-40 | Polysaturated | PC 36:5 | [M-Ac-H]- | 7.9177 | Negative |
| PC | 38 | 3 | 20-40 | Polysaturated | PC 38:3 | [M-CH3]- | 5.3957 | Negative |
| PC | 38 | 4 | 20-40 | Polysaturated | PC 38:4 | [M-Ac-H]- | 8.9086 | Negative |
| PC | 38 | 4 | 20-40 | Polysaturated | PC 38:4 | [M-CH3]- | 6.8794 | Negative |
| PC | 38 | 5 | 20-40 | Polysaturated | PC 38:5 | [M+HCOO]- | 5.5338 | Negative |
| PC | 38 | 5 | 20-40 | Polysaturated | PC 38:5 | [M-Ac-H]- | 7.2567 | Negative |
| PC | 38 | 5 | 20-40 | Polysaturated | PC 38:5 | [M-CH3]- | 6.2257 | Negative |
| PC | 40 | 3 | >= 40 | Polysaturated | PC 40:3 | [M-Ac-H]- | 6.7645 | Negative |
| PC | 44 | 5 | >= 40 | Polysaturated | PC 44:5 | [M-Ac-H]- | 4.638 | Negative |
| PE | 31 | 0 | 20-40 | Saturated | PE 31:0 | [M-H]- | 6.0893 | Negative |
| PE | 31 | 0 | 20-40 | Saturated | PE 31:0 | [M-H]- | 5.4276 | Negative |
| PE | 32 | 1 | 20-40 | Monosaturated | PE 32:1 | [M-H]- | 6.8009 | Negative |
| PE | 33 | 0 | 20-40 | Saturated | PE 33:0 | [M-H]- | 6.058 | Negative |
| PE | 33 | 1 | 20-40 | Monosaturated | PE 33:1 | [M-H]- | 8.7842 | Negative |
| PE | 33 | 2 | 20-40 | Polysaturated | PE 33:2 | [M-H]- | 5.8061 | Negative |
| PE | 34 | 1 | 20-40 | Monosaturated | PE 34:1 | [M-H]- | 6.5124 | Negative |
| PE | 34 | 2 | 20-40 | Polysaturated | PE 34:2 | [M-H]- | 5.9755 | Negative |
| PE | 34 | 3 | 20-40 | Polysaturated | PE 34:3 | [M-H]- | 6.0995 | Negative |
| PE | 34 | 4 | 20-40 | Polysaturated | PE 34:4 | [M-H]- | 6.1568 | Negative |
| PE | 35 | 0 | 20-40 | Saturated | PE 35:0 | [M-H]- | 6.1707 | Negative |
| PE | 35 | 1 | 20-40 | Monosaturated | PE 35:1 | [M-H]- | 6.156 | Negative |
| PE | 36 | 2 | 20-40 | Polysaturated | PE 35:2 | [M-H]- | 6.3526 | Negative |
| PE | 36 | 0 | 20-40 | Saturated | PE 36:0 | [M-H]- | 5.7992 | Negative |
| PE | 36 | 1 | 20-40 | Monosaturated | PE 36:1 | [M-H]- | 5.211 | Negative |
| PE | 36 | 2 | 20-40 | Polysaturated | PE 36:2 | [M-H]- | 6.0408 | Negative |
| PE | 36 | 3 | 20-40 | Polysaturated | PE 36:3 | [M-H]- | 6.1534 | Negative |
| PE | 36 | 4 | 20-40 | Polysaturated | PE 36:4 | [M-H]- | 6.3016 | Negative |
| PE | 36 | 5 | 20-40 | Polysaturated | PE 36:5 | [M-H]- | 5.5197 | Negative |
| PE | 36 | 5 | 20-40 | Polysaturated | PE 36:5 | [M-H]- | 5.0033 | Negative |
| PE | 37 | 4 | 20-40 | Polysaturated | PE 37:4 | [M-H]- | 6.5144 | Negative |
| PE | 38 | 1 | 20-40 | Monosaturated | PE 38:1 | [M-H]- | 7.8065 | Negative |
| PE | 38 | 2 | 20-40 | Polysaturated | PE 38:2 | [M-H]- | 5.4268 | Negative |
| PE | 38 | 4 | 20-40 | Polysaturated | PE 38:4 | [M-H]- | 6.8904 | Negative |
| PE | 38 | 4 | 20-40 | Polysaturated | PE 38:4 | [M-H]- | 8.2925 | Negative |
| PE | 38 | 5 | 20-40 | Polysaturated | PE 38:5 | [M-H]- | 4.8734 | Negative |
| PE | 38 | 6 | 20-40 | Polysaturated | PE 38:6 | [M-H]- | 7.4031 | Negative |
| PE | 39 | 4 | 20-40 | Polysaturated | PE 39:4 | [M-H]- | 6.3665 | Negative |
| PE | 39 | 4 | 20-40 | Polysaturated | PE 39:4 | [M-H]- | 6.6795 | Negative |
| PE | 40 | 1 | >= 40 | Monosaturated | PE 40:1 | [M-H]- | 6.0601 | Negative |
| PE | 40 | 2 | >= 40 | Polysaturated | PE 40:2 | [M-H]- | 8.8833 | Negative |
| PE | 40 | 3 | >= 40 | Polysaturated | PE 40:3 | [M-H]- | 6.0224 | Negative |
| PE | 40 | 4 | >= 40 | Polysaturated | PE 40:4 | [M-H]- | 6.0911 | Negative |
| PE | 40 | 4 | >= 40 | Polysaturated | PE 40:4 | [M-H]- | 8.1884 | Negative |
| PE | 40 | 5 | >= 40 | Polysaturated | PE 40:5 | [M-H]- | 7.2817 | Negative |
| PE | 40 | 5 | >= 40 | Polysaturated | PE 40:5 | [M-H]- | 4.8595 | Negative |
| PE | 40 | 6 | >= 40 | Polysaturated | PE 40:6 | [M-H]- | 7.4341 | Negative |
| PE | 40 | 6 | >= 40 | Polysaturated | PE 40:6 | [M-H]- | 5.4011 | Negative |
| PE | 41 | 1 | >= 40 | Monosaturated | PE 41:1 | [M-H]- | 6.0789 | Negative |
| PE | 42 | 1 | >= 40 | Monosaturated | PE 42:1 | [M-H]- | 6.1699 | Negative |
| PE | 42 | 2 | >= 40 | Polysaturated | PE 42:2 | [M-H]- | 5.7795 | Negative |
| PE | 42 | 2 | >= 40 | Polysaturated | PE 42:2 | [M-H]- | 5.0211 | Negative |
| PE | 42 | 3 | >= 40 | Polysaturated | PE 42:3 | [M-H]- | 5.7044 | Negative |
| PE | 42 | 4 | >= 40 | Polysaturated | PE 42:4 | [M-H]- | 7.8042 | Negative |
| PE | 42 | 4 | >= 40 | Polysaturated | PE 42:4 | [M-H]- | 4.8289 | Negative |
| PE | 44 | 1 | >= 40 | Monosaturated | PE 44:1 | [M-H]- | 5.5822 | Negative |
| PE | 44 | 2 | >= 40 | Polysaturated | PE 44:2 | [M-H]- | 6.0564 | Negative |
| PE | 44 | 2 | >= 40 | Polysaturated | PE 44:2 | [M-H]- | 7.4769 | Negative |
| PE | 44 | 3 | >= 40 | Polysaturated | PE 44:3 | [M-H]- | 5.5733 | Negative |
| PE | 44 | 4 | >= 40 | Polysaturated | PE 44:4 | [M-H]- | 6.0357 | Negative |
| PE | 44 | 5 | >= 40 | Polysaturated | PE 44:5 | [M-H]- | 7.0062 | Negative |
| PG | 26 | 0 | 20-40 | Saturated | PG 26:0 | [M-H]- | 8.6337 | Negative |
| PG | 33 | 0 | 20-40 | Saturated | PG 33:0 | [M-H]- | 4.3032 | Negative |
| PG | 34 | 0 | 20-40 | Saturated | PG 34:0 | [M-H]- | 6.0446 | Negative |
| PG | 34 | 1 | 20-40 | Monosaturated | PG 34:1 | [M-H]- | 5.8146 | Negative |
| PG | 36 | 0 | 20-40 | Saturated | PG 36:0 | [M-H]- | 7.6485 | Negative |
| PG | 36 | 1 | 20-40 | Monosaturated | PG 36:1 | [M-H]- | 6.0361 | Negative |
| PG | 36 | 2 | 20-40 | Polysaturated | PG 36:2 | [M-H]- | 6.2288 | Negative |
| PG | 36 | 3 | 20-40 | Polysaturated | PG 36:3 | [M-H]- | 4.9004 | Negative |
| PG | 38 | 2 | 20-40 | Polysaturated | PG 38:2 | [M-H]- | 6.1591 | Negative |
| PG | 38 | 3 | 20-40 | Polysaturated | PG 38:3 | [M-H]- | 6.7472 | Negative |
| PG | 38 | 4 | 20-40 | Polysaturated | PG 38:4 | [M-H]- | 6.6874 | Negative |
| PG | 38 | 4 | 20-40 | Polysaturated | PG 38:4 | [M-H]- | 6.1507 | Negative |
| PG | 38 | 5 | 20-40 | Polysaturated | PG 38:5 | [M-H]- | 5.3083 | Negative |
| PG | 38 | 6 | 20-40 | Polysaturated | PG 38:6 | [M-H]- | 5.3122 | Negative |
| PG | 40 | 5 | >= 40 | Polysaturated | PG 40:5 | [M-H]- | 5.2987 | Negative |
| PG | 40 | 7 | >= 40 | Polysaturated | PG 40:7 | [M-H]- | 6.0381 | Negative |
| PG | 40 | 8 | >= 40 | Polysaturated | PG 40:8 | [M-H]- | 8.4997 | Negative |
| PG | 42 | 10 | >= 40 | Polysaturated | PG 42:10 | [M-H]- | 5.3136 | Negative |
| PG | 44 | 10 | >= 40 | Polysaturated | PG 44:10 | [M-H]- | 8.8892 | Negative |
| PI | 34 | 0 | 20-40 | Saturated | PI 34:0 | [M-H]- | 8.1915 | Negative |
| PI | 34 | 1 | 20-40 | Monosaturated | PI 34:1 | [M-H]- | 8.1959 | Negative |
| PI | 34 | 1 | 20-40 | Monosaturated | PI 34:1 | [M-H]- | 4.9136 | Negative |
| PI | 36 | 1 | 20-40 | Monosaturated | PI 36:1 | [M-H]- | 7.4471 | Negative |
| PI | 36 | 2 | 20-40 | Polysaturated | PI 36:2 | [M-H]- | 6.1974 | Negative |
| PI | 36 | 4 | 20-40 | Polysaturated | PI 36:4 | [M-H]- | 8.9451 | Negative |
| PI | 37 | 3 | 20-40 | Polysaturated | PI 37:3 | [M-H]- | 7.4347 | Negative |
| PI | 38 | 2 | 20-40 | Polysaturated | PI 38:2 | [M-H]- | 8.5302 | Negative |
| PI | 38 | 2 | 20-40 | Polysaturated | PI 38:2 | [M-H]- | 7.0523 | Negative |
| PI | 38 | 3 | 20-40 | Polysaturated | PI 38:3 | [M-H]- | 5.9205 | Negative |
| PI | 38 | 4 | 20-40 | Polysaturated | PI 38:4 | [M-H]- | 6.6023 | Negative |
| PI | 38 | 4 | 20-40 | Polysaturated | PI 38:4 | [M-H]- | 6.0421 | Negative |
| PI | 38 | 5 | 20-40 | Polysaturated | PI 38:5 | [M-H]- | 5.4082 | Negative |
| PI | 40 | 3 | >= 40 | Polysaturated | PI 40:3 | [M-H]- | 6.6007 | Negative |
| PI | 40 | 4 | >= 40 | Polysaturated | PI 40:4 | [M-H]- | 7.4201 | Negative |
| PI | 40 | 4 | >= 40 | Polysaturated | PI 40:4 | [M-H]- | 4.9541 | Negative |
| PI | 40 | 5 | >= 40 | Polysaturated | PI 40:5 | [M-H]- | 6.1728 | Negative |
| PI | 40 | 6 | >= 40 | Polysaturated | PI 40:6 | [M-H]- | 7.9299 | Negative |
| plasmenyl-PE | 34 | 1 | 20-40 | Monosaturated | plasmenyl-PE 34:1 | [M-H]- | 7.9255 | Negative |
| plasmenyl-PE | 34 | 3 | 20-40 | Polysaturated | plasmenyl-PE 34:3 | [M-H]- | 6.8808 | Negative |
| plasmenyl-PE | 36 | 5 | 20-40 | Polysaturated | plasmenyl-PE 36:5 | [M-H]- | 7.9448 | Negative |
| plasmenyl-PE | 38 | 4 | 20-40 | Polysaturated | plasmenyl-PE 38:4 | [M-H]- | 5.33 | Negative |
| plasmenyl-PE | 38 | 5 | 20-40 | Polysaturated | plasmenyl-PE 38:5 | [M-H]- | 2.265 | Negative |
| plasmenyl-PE | 38 | 6 | 20-40 | Polysaturated | plasmenyl-PE 38:6 | [M-H]- | 5.7658 | Negative |
| plasmenyl-PE | 40 | 6 | >= 40 | Polysaturated | plasmenyl-PE 40:6 | [M-H]- | 4.3258 | Negative |
| plasmenyl-PE | 42 | 6 | >= 40 | Polysaturated | plasmenyl-PE 42:6 | [M-H]- | 8.7676 | Negative |
| PS | 32 | 0 | 20-40 | Saturated | PS 32:0 | [M-H]- | 7.9401 | Negative |
| PS | 34 | 0 | 20-40 | Saturated | PS 34:0 | [M-H]- | 8.8672 | Negative |
| PS | 34 | 0 | 20-40 | Saturated | PS 34:0 | [M-H]- | 7.8048 | Negative |
| PS | 34 | 1 | 20-40 | Monosaturated | PS 34:1 | [M-H]- | 6.1246 | Negative |
| PS | 34 | 2 | 20-40 | Polysaturated | PS 34:2 | [M-H]- | 8.3153 | Negative |
| PS | 35 | 2 | 20-40 | Polysaturated | PS 35:2 | [M-H]- | 7.02 | Negative |
| PS | 35 | 2 | 20-40 | Polysaturated | PS 35:2 | [M-H]- | 5.3599 | Negative |
| PS | 36 | 1 | 20-40 | Monosaturated | PS 36:1 | [M-H]- | 6.8078 | Negative |
| PS | 36 | 2 | 20-40 | Polysaturated | PS 36:2 | [M-H]- | 7.1588 | Negative |
| PS | 36 | 2 | 20-40 | Polysaturated | PS 36:2 | [M-H]- | 5.2768 | Negative |
| PS | 36 | 3 | 20-40 | Polysaturated | PS 36:3 | [M-H]- | 6.7154 | Negative |
| PS | 37 | 3 | 20-40 | Polysaturated | PS 37:3 | [M-H]- | 8.3769 | Negative |
| PS | 37 | 4 | 20-40 | Polysaturated | PS 37:4 | [M-H]- | 6.7076 | Negative |
| PS | 37 | 5 | 20-40 | Polysaturated | PS 37:5 | [M-H]- | 4.7751 | Negative |
| PS | 38 | 1 | 20-40 | Monosaturated | PS 38:1 | [M-H]- | 6.0809 | Negative |
| PS | 38 | 2 | 20-40 | Polysaturated | PS 38:2 | [M-H]- | 8.2017 | Negative |
| PS | 38 | 3 | 20-40 | Polysaturated | PS 38:3 | [M-H]- | 5.4039 | Negative |
| PS | 38 | 4 | 20-40 | Polysaturated | PS 38:4 | [M-H]- | 6.2284 | Negative |
| PS | 38 | 4 | 20-40 | Polysaturated | PS 38:4 | [M-H]- | 5.3187 | Negative |
| PS | 38 | 5 | 20-40 | Polysaturated | PS 38:5 | [M-H]- | 5.7716 | Negative |
| PS | 39 | 5 | 20-40 | Polysaturated | PS 39:5 | [M-H]- | 5.4336 | Negative |
| PS | 40 | 1 | >= 40 | Monosaturated | PS 40:1 | [M-H]- | 2.3537 | Negative |
| PS | 40 | 2 | >= 40 | Polysaturated | PS 40:2 | [M-H]- | 6.0362 | Negative |
| PS | 40 | 4 | >= 40 | Polysaturated | PS 40:4 | [M-H]- | 7.8386 | Negative |
| PS | 40 | 5 | >= 40 | Polysaturated | PS 40:5 | [M-H]- | 7.8146 | Negative |
| PS | 40 | 6 | >= 40 | Polysaturated | PS 40:6 | [M-H]- | 6.1008 | Negative |
| PS | 40 | 6 | >= 40 | Polysaturated | PS 40:6 | [M-H]- | 6.8676 | Negative |
| PS | 40 | 7 | >= 40 | Polysaturated | PS 40:7 | [M-H]- | 6.741 | Negative |
| PS | 41 | 1 | >= 40 | Monosaturated | PS 41:1 | [M-H]- | 5.7643 | Negative |
| PS | 42 | 1 | >= 40 | Monosaturated | PS 42:1 | [M-H]- | 7.8746 | Negative |
| PS | 42 | 2 | >= 40 | Polysaturated | PS 42:2 | [M-H]- | 6.1953 | Negative |
| PS | 42 | 2 | >= 40 | Polysaturated | PS 42:2 | [M-H]- | 9.1172 | Negative |
| PS | 42 | 4 | >= 40 | Polysaturated | PS 42:4 | [M-H]- | 5.7374 | Negative |
| PS | 42 | 4 | >= 40 | Polysaturated | PS 42:4 | [M-H]- | 5.4223 | Negative |
| PS | 42 | 6 | >= 40 | Polysaturated | PS 42:6 | [M-H]- | 6.0955 | Negative |
| PS | 44 | 4 | >= 40 | Polysaturated | PS 44:4 | [M-H]- | 6.8166 | Negative |
| PS | 44 | 5 | >= 40 | Polysaturated | PS 44:5 | [M-H]- | 6.7984 | Negative |
| PS | 44 | 7 | >= 40 | Polysaturated | PS 44:7 | [M-H]- | 7.3737 | Negative |
